# The HARE chip for efficient time-resolved serial synchrotron crystallography

**DOI:** 10.1107/S1600577520000685

**Published:** 2020-02-27

**Authors:** Pedram Mehrabi, Henrike M. Müller-Werkmeister, Jan-Philipp Leimkohl, Hendrik Schikora, Jelena Ninkovic, Silvia Krivokuca, Ladislav Andriček, Sascha W. Epp, Darren Sherrell, Robin L. Owen, Arwen R. Pearson, Friedjof Tellkamp, Eike C. Schulz, R. J. Dwayne Miller

**Affiliations:** a Department for Atomically Resolved Dynamics, Max-Planck-Institute for Structure and Dynamics of Matter, Luruper Chaussee 149, 22761 Hamburg, Germany; b Institute of Chemistry – Physical Chemistry, University of Potsdam, Karl-Liebknecht-Strasse 24-25, 14476 Potsdam-Golm, Germany; c Scientific Support Unit Machine Physics, Max-Planck-Institute for Structure and Dynamics of Matter, Luruper Chaussee 149, 22761 Hamburg, Germany; d Halbleiterlabor der Max-Planck-Gesellschaft, Otto-Hahn-Ring 6, D-81739 Munich, Germany; e Diamond Light Source, Harwell Science and Innovation Campus, Didcot OX11 0DE, UK; f Department of Physics, Universität Hamburg, Jungiusstrasse 9, 20355 Hamburg, Germany; g Departments of Chemistry and Physics, University of Toronto, 80 St George Street, Toronto, Ontario M5S 3H6, Canada

**Keywords:** data collection, sample delivery, fixed-target serial synchrotron crystallography, time-resolved crystallography, HARE, LAMA

## Abstract

Serial synchrotron crystallography (SSX) is an emerging technique for static and time-resolved protein structure determination. Using specifically patterned silicon chips for sample delivery, the ‘hit-and-return’ (HARE) protocol allows for efficient time-resolved data collection. The specific pattern of the crystal wells in the HARE chip provides direct access to many discrete time points. HARE chips allow for optical excitation as well as on-chip mixing for reaction initiation, making a large number of protein systems amenable to time-resolved studies. Loading of protein microcrystals onto the HARE chip is streamlined by a novel vacuum loading platform that allows fine-tuning of suction strength while maintaining a humid environment to prevent crystal dehydration. To enable the widespread use of time-resolved serial synchrotron crystallography (TR-SSX), detailed technical descriptions of a set of accessories that facilitate TR-SSX workflows are provided.

## Abstract 2

The HARE chip and a toolbox of helpful instrumentation for time-resolved serial synchrotron crystallography are presented.

## 1. Introduction   

Time-resolved crystallography is one of the few techniques that can provide simultaneous insight into structure and dynamics with near-atomic resolution (Moffat, 1989 ▸, 1998 ▸; Pai, 1992 ▸). This method has experienced a renaissance since the advent of high-brilliance X-ray free-electron laser (XFEL) sources. XFELs triggered the development of serial diffraction data-collection methods because a single high-intensity FEL pulse can lead to the destruction of the crystal under study (Chapman, 2019 ▸; Chapman *et al.*, 2011 ▸). Serial approaches have the additional advantage of avoiding many of the difficulties traditionally associated with time-resolved studies of single crystals, *i.e.* reaction initiation, radiation damage and signal-to-noise. Taking advantage of the ultrashort time-resolutions accessible at XFELs, many of the initial FEL studies have probed sub-picosecond timescales using liquid-jet delivery systems (Martin-Garcia *et al.*, 2016 ▸; Kupitz *et al.*, 2017 ▸, 2014 ▸; Lee *et al.*, 2018 ▸; Schlichting, 2015 ▸; Chapman, 2019 ▸; Tenboer *et al.*, 2014 ▸; Barends *et al.*, 2015 ▸). Liquid jets brought new challenges: collecting thousands of still diffraction patterns mandates high-velocity crystal exchange, which initially resulted in large sample consumption and waste. This made studying scarce, expensive or difficult to crystallize systems unfeasible and led to the development of high-viscosity extruders and fixed-target solutions with greatly reduced sample consumption (Suga *et al.*, 2019 ▸; Hunter *et al.*, 2014 ▸; Weierstall *et al.*, 2014 ▸; Martiel *et al.*, 2019 ▸; Grünbein & Nass Kovacs, 2019 ▸; Zarrine-Afsar *et al.*, 2012 ▸; Mueller *et al.*, 2015 ▸).

Serial crystallographic experiments can also be conducted at synchrotrons (serial synchrotron crystallography, SSX), approximating the low-damage XFEL structures by using the serial data-collection approach to minimize dose accumulation (Roedig *et al.*, 2016 ▸; Gati *et al.*, 2014 ▸; Stellato *et al.*, 2014 ▸; Chapman *et al.*, 2014 ▸; Ebrahim, Moreno-Chicano *et al.*, 2019 ▸; Owen *et al.*, 2017 ▸; Weinert *et al.*, 2017 ▸). Initially, SSX experiments were conducted using a simple mesh-screening approach with microcrystals randomly positioned and frozen in a loop, or by pushing a crystal suspension through a capillary in the X-ray beam (Gati *et al.*, 2014 ▸; Stellato *et al.*, 2014 ▸). Following these initial proof-of-principle experiments, hybrid methods such as tape drive approaches as well as multiple fixed-target solutions have been developed at synchrotrons for both cryo- and room-temperature crystallography (Martiel *et al.*, 2019 ▸). These methods present an alternative to liquid-jet-based sample-delivery systems and have the advantage of increased sample integrity, reduced sample consumption and hit-rate optimization (Roedig *et al.*, 2015 ▸, 2016 ▸, 2017 ▸; Martiel *et al.*, 2019 ▸; Oghbaey *et al.*, 2016 ▸; Zarrine-Afsar *et al.*, 2012 ▸, 2010 ▸; Mueller *et al.*, 2015 ▸; Sherrell *et al.*, 2015 ▸; Owen *et al.*, 2017 ▸). In combination with a fast accurate translation-stage system, silicon-based chips, such as those described here, deliver high crystal-well densities on the chip, high hit-rates and allow serial sampling of ∼100 000 crystal wells per hour at room temperature (Owen *et al.*, 2017 ▸; Wierman *et al.*, 2019 ▸; Sherrell *et al.*, 2015 ▸; Oghbaey *et al.*, 2016 ▸; Ebrahim, Moreno-Chicano *et al.*, 2019 ▸; Ebrahim, Appleby *et al.*, 2019 ▸). Using next-generation light sources and detectors allows the collection of multiple images per crystal well, which significantly increases the throughput (Ebrahim, Appleby *et al.*, 2019 ▸; Tolstikova *et al.*, 2019 ▸). We note, however, that the lifetime of the crystals is still limited by the applicable X-ray dose and therefore the number of images that can be obtained.

While XFELs are uniquely suited to provide insight into ultrafast dynamics on the femtosecond scale, metastable reaction intermediates of most enzyme mechanisms occur on the microsecond to second time domain and this can conveniently be addressed at third- and fourth-generation synchrotron sources (Schlichting, 2015 ▸; Chapman, 2019 ▸; Bar-Even *et al.*, 2011 ▸; Weinert *et al.*, 2019 ▸; Schulz *et al.*, 2018 ▸; Mehrabi, Schulz, Dsouza *et al.*, 2019 ▸; Mehrabi, Schulz, Agthe *et al.*, 2019 ▸). However, a significant problem when utilizing serial approaches for these longer delay times is the inevitable increase in overall data-collection time for a standard pump–delay–probe experiment, which makes data acquisition at long time delays impractical within a standard 24 hour beam time. We recently demonstrated that this problem can be alleviated with a ‘hit-and-return’ (HARE) method. HARE allows collection of sufficient SSX data for approximately one time point per hour irrespective of the delay time (Schulz *et al.*, 2018 ▸). In brief, the data-collection sequence begins by probing then pumping a set number of crystal wells which correspond to a specific HARE number. The initial probe image serves as a reference. The system returns to the initial crystal well, then probes the pumped wells again to obtain the pumped (delayed) image. As the translation time between all wells is identical, all crystal wells have the same desired time delay. The attainable HARE numbers are dictated by the pattern of crystal wells on the chip. We initially demonstrated this approach by using a photocaged substrate and laser-triggered reaction initiation to follow an enzymatic reaction over almost 30 s (Mehrabi, Schulz, Dsouza *et al.*, 2019 ▸). We also combined the HARE approach with our on-chip mixing technique LAMA (Mehrabi, Schulz, Agthe *et al.*, 2019 ▸). To be most efficient, the HARE approach requires a specific pattern of the crystal wells on the chip. We have therefore developed a dedicated HARE chip design that is described in detail here.

A further important aspect in the preparation of any fixed-target SSX experiment is mounting the crystals on the chip and achieving a high fraction of loaded crystal wells. Fragile protein crystals can suffer from physical stress as well as dehydration during the loading process. To mitigate these problems, we have recently substantially improved our previously described crystal-loading pipeline (Mueller *et al.*, 2015 ▸; Oghbaey *et al.*, 2016 ▸). The new chip-loading platform, presented here, ensures humidity control and limits the force acting on the individual crystals in order to minimize detrimental effects during the loading process while maintaining compatibility with a variety of different crystallization mother liquors.

We have already successfully applied the tools and protocols described above to carry out time-resolved serial synchrotron crystallography (TR-SSX) experiments for three different proteins (Mehrabi, Schulz, Dsouza *et al.*, 2019 ▸; Schulz *et al.*, 2018 ▸; Mehrabi, Schulz, Agthe *et al.*, 2019 ▸). In this report, we describe the HARE chip and provide details of our current best-practice protocols for chip maintenance and sample loading. These protocols include simple cheap off-the-shelf options as well as more comprehensive bespoke solutions. We hope this is a useful resource that will enable widespread use of TR-SSX at synchrotron beamlines worldwide.

## 2. Methods   

### 2.1. Protein crystallization   

Protein crystallization was conducted as described previously (Mehrabi, Schulz, Agthe *et al.*, 2019 ▸).

Xylose isomerase crystals were obtained from Hampton Research (HR7-102) and dissolved in water. Redissolved crystals were concentrated to 80 mg ml^−1^ in xylose isomerase buffer (10 m*M* Hepes/NaOH pH 7.5). To displace any remaining Tris-HCl from the storage condition the concentrated protein solution was re-diluted and concentrated again for nine consecutive times. Vacuum-induced crystallization in XI crystallization buffer [35%(*w*/*v*) PEG3350, 200 m*M* lithium sulfate and 10 m*M* Hepes/NaOH pH 7.5] yielded the microcrystals.

### 2.2. Serial X-ray diffraction experiments   

All serial X-ray diffraction experiments were conducted at room temperature at EMBL beamline P14-2 at the PETRA III synchrotron at DESY, Hamburg (https://www.embl-hamburg.de/services/mx/P14_EH2/). For sample exchange the chips were mounted on the translation-stage holders as described below and connected to the SmarAct translation stages as described previously (Schulz *et al.*, 2018 ▸; Sherrell *et al.*, 2015 ▸).

## 3. Results   

### 3.1. The HARE chip for TR-SSX   

In contrast to first-generation chip designs, the HARE chips have an increased outer dimension of 30 mm by 30 mm to accommodate a higher number of crystal wells per chip (Mueller *et al.*, 2015 ▸; Zarrine-Afsar *et al.*, 2012 ▸; Oghbaey *et al.*, 2016 ▸). This is the same form factor as used in the Oxford photochip, which has already been described elsewhere (Ebrahim, Moreno-Chicano *et al.*, 2019 ▸; Ebrahim, Appleby *et al.*, 2019 ▸). The HARE chip design also allows for smaller crystal sizes and higher crystal-well densities. However, the main design goal of the HARE chip was to simplify and streamline time-resolved pump–probe applications. To this end the HARE-chip design is fourfold symmetric, simplifying motion control and chip alignment.

#### 3.1.1. HARE chip design   

**Figure 1:**
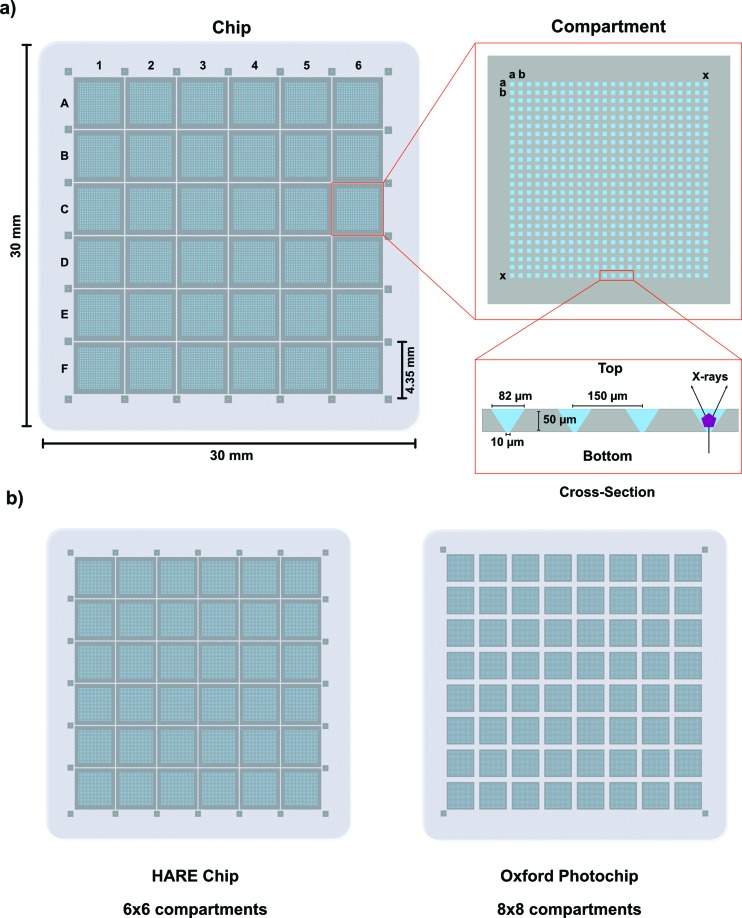
Design of the HARE chip. (*a*) Left: overview of the HARE chip, consisting of 6 × 6 compartments (A1–F6). Top right: an individual compartment is magnified showing the individual features *aa*–*xx*. Bottom right: A cross section of four microwells displays the dimensions. (*b*) Side-by-side comparison of the HARE chip with the Oxford photochip.

The HARE chip is divided into 6 × 6 compartments (A1–F6) of 3.6 mm × 3.6 mm separated by 900 µm gaps in each direction. For best implementation of the HARE approach, each compartment is subdivided into 24 × 24 crystal wells, resulting in 20 736 crystal wells per chip. This provides a high composite number of crystal wells per row (24), allowing maximum flexibility in specific delay times that can be addressed without any other modification of the experimental parameters, without making the chip too fragile (36 wells per row is too many) (Schulz *et al.*, 2018 ▸). The pitch of the crystal wells is 150 µm in both lateral directions. The nominal dimensions of the square crystal wells are thus 82 µm on the top side and 10 µm on the bottom side (Figs. 1 ▸ and S1 in the supporting information). The compartment area is lined with fiducial marks etched into the silicon between the junctions of the outer compartments at a pitch of 4350 µm. The nominal dimensions of the fiducial marks are 86 µm on the top and 20 µm on the bottom and they are situated 850 µm from the centre of the adjacent aperture. Detailed CAD drawings of the HARE chip, which can be used for mask development, can be found in the supporting information (supporting material 1).

#### 3.1.2. Production of the HARE chips   

**Figure 2:**
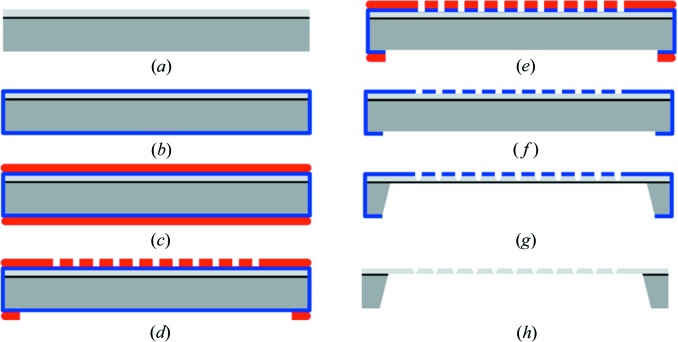
Sequence of the HARE chip production. The HARE chips are produced in eight consecutive protection and etching steps described in detail in the text. Dark/light grey = monocrystalline silicon of 450 and 50 µm thickness, respectively. Black = 300 nm layer of SiO_2_. Blue = 250 nm thermal oxide layer. Red = photoresist.

The HARE chips are manufactured from conventional double side polished silicon-on-insulator wafers and are produced as follows. (*a*) The production process sequence starts with two layers of monocrystalline silicon in [100] orientation. A 50 µm-thick top layer and 450 µm-thick bottom layer are separated by a thin (300 nm) layer of thermally grown SiO_2_ (BOX, bonding oxide) [Fig. 2 ▸(*a*)]. (*b*) A thermal oxide of 250 nm is grown on the wafers which acts as the hard mask for the etching in the following steps [Fig. 2 ▸(*b*)]. (*c*) Photoresist is spun on the oxidized wafers on both sides to structure the hard mask [Fig. 2 ▸(*c*)]. (*d*) The photoresist layer is exposed through a glass mask with UV light and the exposed regions on the wafer are then removed in a photoresist development step [Fig. 2 ▸(*d*)]. (*e*) The oxide is wet chemically removed from the wafers in the open regions of the photoresist [Fig. 2 ▸(*e*)] and (*f*) the photoresist layer is stripped [Fig. 2 ▸(*f*)]. (*g*) The wafers with the structured oxide layer are then immersed in a wet chemical bath with TMAH (tetra­methyl ammonium hydroxide). The exposed silicon is dissolved while the oxide acts as a hard mask for the etching and the BOX acts as an etch stop for the process [Fig. 2 ▸(*g*)]. In this way the etch process is self-limiting and the etching of both sides can be done in one single step. The lateral etch rate is suppressed by the anisotropic etch rates of the TMAH solution. (*h*) As a last step, the hard mask including the BOX is removed by immersion into hydrofluoric acid [Fig. 2 ▸(*h*)] after which the chips can be cut out of the wafer.

### 3.2. The chip sample holder   

**Figure 3:**
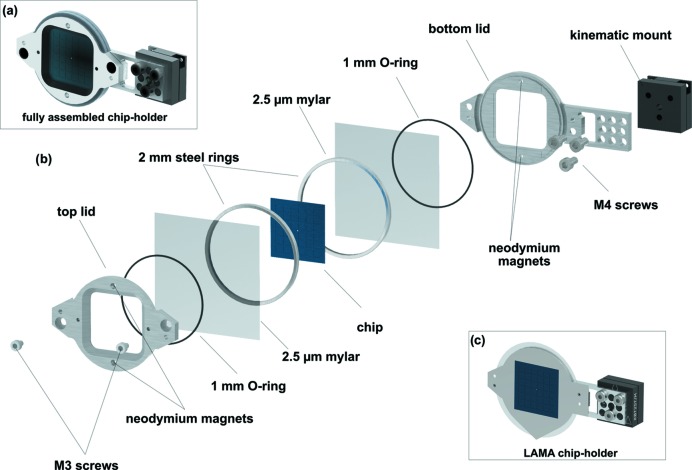
The chip sample holder. The multi-component sample holder is important both for efficient alignment and translation of the chip in the X-ray beam and, for LAMA experiments, to maintain a humid environment for the protein crystals. (*a*) A fully assembled chip holder containing a HARE chip. (*b*) All components of a chip holder in an exploded view. (*c*) A fully assembled chip holder with the tear-shaped cover for LAMA applications.

To mount the chips onto the translation stages used for SSX (Sherrell *et al.*, 2015 ▸), new sample holders were designed to accommodate the larger chip dimensions (Fig. 3 ▸). To simplify the mounting of chips onto the stages the sample holders include half of a kinematic mount (KBT25T/M, Thorlabs Inc.), while the other half (KBB25/M, Thorlabs Inc.) is permanently attached to the stages. This allows the holder to easily snap into position during sample exchange. To prevent dehydration of the protein crystals during data collection, the chips were sealed with 2.5 µm Mylar foils, which were integrated into the holder. Wrinkling of the Mylar foils was prevented by stretching them across the two half holders using tight-fitting circular rings. The two halves of the holder were equipped with neodymium magnets of opposite polarity to simplify the alignment and closing of the holder. After the chip was placed on the Mylar covered holder, the lid was fixed with two M3 screws. We found that 66 mm circular cut Mylar sheets (2.5 µm thickness) (Chemplex Industries Inc. USA) are much more convenient for sealing the chips than cutting foils to size from a roll or larger sheets.

Crystal dehydration can also be prevented by humidified environment (Sanchez-Weatherby *et al.*, 2009 ▸). For LAMA applications, where a small droplet of ligand is added to each crystal on the chip, the top half of the holder is replaced with a tear-shaped stainless steel cover with no Mylar foil and the crystals are prevented from dehydrating using a humidified gas stream. To this end, a constant flow of humid air is generated by directing the humidity stream from a low-cost room humidifier via a simple rubber hose. The tear shape of the LAMA holder channels any condensation from the humidified gas stream away from the surface of the chip and holder via gravity and can be collected in a simple plastic dish, thereby preventing leakage onto nearby electronic devices (Mehrabi, Schulz, Agthe *et al.*, 2019 ▸). The supporting information includes CAD drawings of the chip holder and the tear-shaped cover extension required for LAMA (supporting material 2 and 3).

### 3.3. Chip maintenance   

#### 3.3.1. Cleaning   

**Figure 4:**
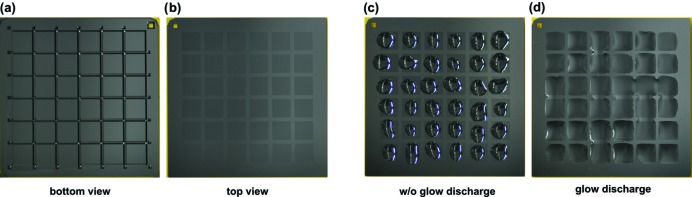
The effect of glow discharging on HARE chips. Photographs of HARE chips: (*a*) bottom view showing the compartment support bars, (*b*) top view showing the 36 compartments, (*c*) top view showing 3.5 µl of ddH_2_O loaded onto each compartment before glow discharging clearly forms droplets, and (*d*) top view showing 3.5 µl of ddH_2_O loaded onto each compartment after glow discharging evenly spreads out across each compartment and avoids the hydro­philic areas in between.

The simplest method to clean the chips is extensive incubation in water, which dissolves most residues over time. However, the chips can be more effectively cleaned after each data collection using the following protocol. Directly after use the chips are incubated in a solution of 1%(*w*/*v*) TERGAZYME (Sigma–Aldrich) or a 1 *M* HCl solution for 1 h. Subsequently, the chips are rinsed under running hot tap water and then washed with ddH_2_O. Finally, the chips are washed with iso­propanol before being left to dry on the bench for a few hours. To speed up drying, chips can be placed in a custom-built drying station, attached to the pressured air or nitro­gen gas feed usually found at synchrotron beamlines, and exposed to pressurized gas for ∼5 min [Fig. 4 ▸(*a*)]. The information needed to print the drying station using a 3D printer is available in supporting material 4.

Should they be contaminated with stubborn residue, chips can be comprehensively cleaned using hot piranha solution [H_2_O_2_(30% *w*/*w*):H_2_SO_4_ in a ratio of 1:2.5 at 100°C], which removes all organic contaminants and leaves the chip surface hydro­philic. We note that piranha solution is extremely caustic and should only be used with appropriate safety precautions and personal protective equipment.

#### 3.3.2. Glow discharging   

Alteration of the surface charge allows for a more even wetting of the chips, improving crystal-loading homogeneity. As an alternative to chemical treatments, a convenient solution to generate hydro­philic surfaces is glow discharging or plasma cleaning. For a simple surface treatment of the whole chip, individual chips can simply be placed in a glow discharger (Balzers CTA 010, Balzers Union, Switzerland) one at a time. Chips are then glow discharged for 60 s at 35 mA. To allow a selective wetting of the crystal-well containing compartments on the chip we designed a custom chamber which can hold four chips covered with laser-cut Kapton masks. These masks selectively cover all non-compartment areas of the chips. Closing the chamber presses the Kapton mask onto the chip. The selective wetting of the compartment areas leads to a more homogeneous liquid-layer distribution on the compartments, while the bars between compartments are more hydro­phobic and repel the crystal slurry (Figs. 4 ▸ and 5 ▸). Information needed to reproduce the glow-discharge holder using a 3D printer is available in supporting material 5.

#### 3.3.3. Storage   

**Figure 5:**
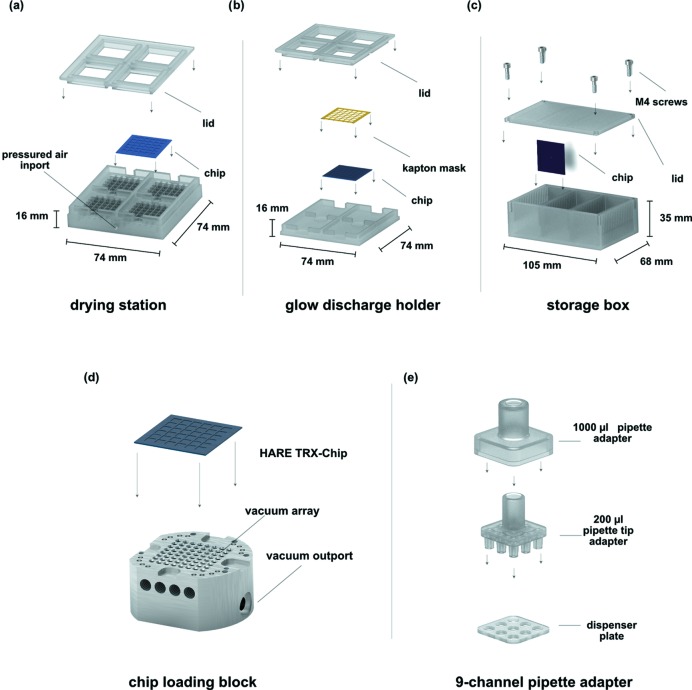
Chip maintenance and loading solutions. (*a*) The chip-drying station for quick drying of the chips after cleaning. (*b*) The glow-discharge holder, which holds Kapton masks and chips together during the glow-discharge process. (*c*) The storage box for safe and easy transportation of the fragile chips. (*d*) The chip-loading block allows efficient loading of crystalline slurries by providing a convenient vacuum seal between the polished stainless steel surface and the chip. (*e*) The nine-channel pipette adapter connects a conventional 1000 µl micropipette with nine 200 µl pipette tips. This allows for an increased homogeneity of the dispensed crystal slurry on the chip surface.

Chips can be simply stored in small plastic membrane boxes that are cheaply available (*i.e.* Agar Scientific Ltd, UK). However, we found that to streamline handling during beam time, increase chip longevity, and safeguard the delicate chips during transport and storage, a custom storage box is extremely helpful. In our design, as in a microscope ‘slide box’, the chips are stored vertically in three arrays of 19 chips each. In comparison with membrane boxes, this minimizes storage space and allows for safe and convenient transport of a large number of chips to the beamline (Fig. 5 ▸). Information to reproduce the storage box using a 3D printer is available in supporting material 6.

### 3.4. The chip-loading platform   

Crystal loading onto the chips can be simply achieved by applying a slurry of crystals using a pipette and a laboratory vacuum pump to suck the crystals into the crystal wells via a chip-loading block (Mueller *et al.*, 2015 ▸; Oghbaey *et al.*, 2016 ▸). However, without proper control of both the vacuum and local humidity, crystal damage as a result of physical stress and/or dehydration is possible. A cheap, off-the-shelf solution is to use a dial gauge to control the vacuum pressure and to place the whole mounting assembly into a disposable humidity tent (SoloLab; solocontainment, UK) containing a standard home room humidifier. However, this does not allow the vacuum pressure or the humidity surrounding the crystals to be precisely maintained. We have therefore developed a low-cost humidity hood with an automatic humidity feedback control (Rotronic Hydro­flex, Ettlingen, Germany), which provides a more accurate control over the humidity, as well as a vacuum control unit. We will also describe a custom-built multi-channel pipette adapter, which can further simplify chip loading.

#### 3.4.1. Chip-loading block   

Our original chip-loading solution utilized an aluminium block with a single outlet port connected to a vacuum pump. The top of the block was covered with a silicon slide to form a vacuum-tight seal with the chip (Oghbaey *et al.*, 2016 ▸). The new chip-loading block design presented here consists of a polished stainless steel block that is mounted on a stainless steel base for stability (Fig. 5 ▸). The flatness of the polished surface allows a tight seal between the chip and the loading block. To allow a more homogeneous distribution of the vacuum, the loading block has an 8 × 8 array of holes that funnel into a single outlet port that connects to the vacuum control device. Chips are held in place prior to application of the vacuum by steel spring clips. All tubing connections are via push-in fittings (Sang-A, Landefeld, Germany). Supporting material 7 contains detailed CAD drawings of the chip-loading block that should allow for reproduction in local machine shops.

#### 3.4.2. A multi-channel pipette adapter   

Even loading of crystal slurry onto the chip is crucial for all SSX experiments. This can simply be carried out using a single-channel pipette to distribute the crystal slurry over the chip surface. However, to improve the homogeneity of the crystal slurry distribution over the chip we have developed a nine-channel pipette adapter (Fig. 5 ▸). The adapter is designed to fit on a standard 1000 µl micropipette and to connect this to an array of nine 200 µl pipette tips. A dispenser plate glued into the 1000 µl pipette adapter allows for convenient ejection of used pipette tips. For chip loading a crystal slurry is transferred to a typical 96-well PCR plate prior to loading of the nine-channel pipette. The information needed to reproduce the nine-channel pipette adapter using a 3D printer is available in supporting material 8.

#### 3.4.3. Vacuum control unit   

**Figure 6:**
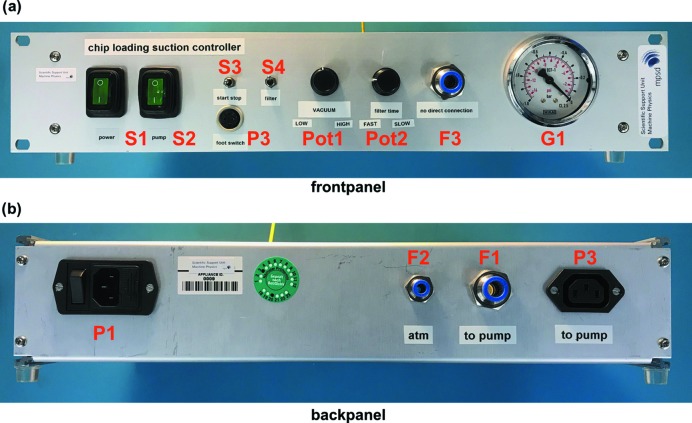
The vacuum control unit. (*a*) View of the front panel. From left to right: mains switches for the control unit (S1) and the pump (S2), a connector for the foot paddle (P3), control switches for the vacuum (S3), and filter insertion to control the vacuum ramp up/down (S4). Vacuum level (Pot1) and filter insertion rate (Pot2) can be adjusted using analogue potentiometers. An outlet port with push-in fitting (F3) connects the vacuum pump to the chip-loading block and an analogue pressure gauge (G1) aids in setting the desired backpressure. (*b*) View of the back panel. From left to right: 230 V power socket (P1), vent to atmosphere (F2), inlet port with push-in fitting connecting to the vacuum pump (F1) and membrane pump power feed-through (P2).

The vacuum control unit we have developed allows us to set specific vacuum levels, monitored by an analogue pressure gauge at the front of the device, as well as to control pressure ramps during the chip-loading process (Fig. 5 ▸). An analogue electronics board (PCB) allows gentle ramping up and down of control voltages for the vacuum controller which, in turn, provides a corresponding vacuum level. The vacuum is generated by a 230 V membrane pump (Pfeiffer MVP-040–2, Germany). The vacuum can be switched on or off via a manual switch on the control panel, or by using a convenient foot pedal, but also allows for continuous pumping (Fig. 6 ▸). For new systems we typically start to test vacuum levels between 0.3 and 0.5 bar. Refer to supporting material 9 for detailed CAD drawings of the vacuum control unit.

#### 3.4.4. Humidity hood   

**Figure 7:**
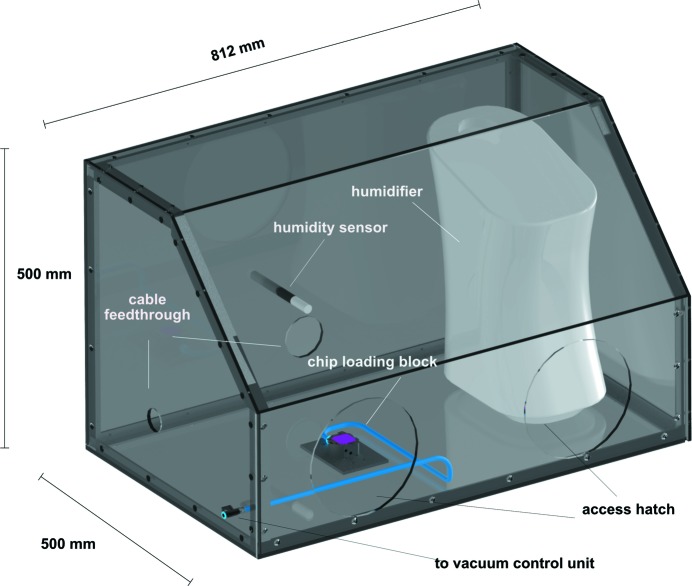
The humidity control hood. An 3D schematic showing the humidity control hood, with the humidifier, humidity sensor and chip-loading block inside.

To prevent dehydration of the crystals during loading and transfer of the loaded chips into the holders, the vacuum loading block is placed in a humidity control hood made of plexiglass (Fig. 7 ▸). Humidity is maintained at the desired level by connecting a low-cost commercial room humidifier (Honeywell, USA) to a humidity control sensor (Testo, Germany) via a proportional integral derivative controller unit (KR3, Ascon Technologic, Italy). During loading the relative humidity in the hood is typically kept at 85%. Refer to supporting material 10 for detailed CAD drawings of the humidity hood and the humidity control unit.

### 3.5. Chip-loading protocols   

#### 3.5.1. Determining sample concentration for microcrystals   

**Figure 8:**
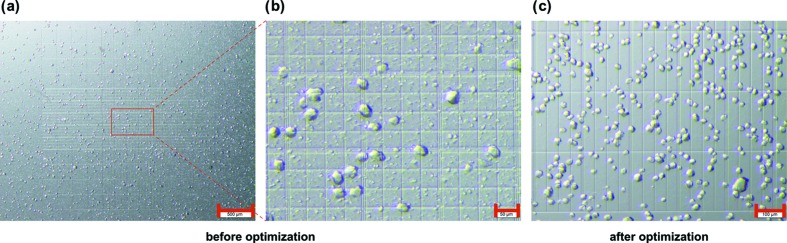
Crystal size and homogeneity estimation using a Neubauer counting chamber. Xylose isomerase microcrystals are shown before and after optimization of the batch crystallization process for improved size homogeneity. Before optimization (*a*),(*b*) the sample shows high crystal size heterogeneity. While one of the strengths of the chip technology lies in its tolerance of variable crystal sizes, a homogeneous crystal size distribution is advantageous for both homogeneous reaction initiation in time-resolved experiments and for stability of scaling during data processing. (*a*) Overview of xylose isomerase microcrystals in a Neubauer cell-counting chamber (the scale bar is 500 µm). (*b*) Close-up view of the indicated area (the scale bar is 50 µm). (*c*) Xylose isomerase sample after optimization of the crystal size homogeneity (the scale bar is 100 µm). This crystal concentration (∼2.88 × 10^6^ ml^−1^) should be diluted by a factor of two for TR-SSX experiments with the vacuum loading approach (described below), to avoid overloading the chip.

In order to prevent under- or over-loading of the chip the concentration of the protein microcrystals should be assessed. We note that, for the chips described here, crystals should be ≥10 µm to avoid them flowing straight through the chip holes. A convenient and cost-effective way to quickly assess the concentration of protein microcrystal solutions during beam times is to use a simple benchtop light microscope and a Neubauer cell-counting chamber (NanoEnTek, Korea) into which 10 µl of crystal slurry are deposited. At 10×–40× magnification it is possible to not only assess the concentration of the protein microcrystals but also to assess the homogeneity of the crystal size distribution (Fig. 8 ▸). In our hands, crystal concentrations of 1 × 10^6^ crystals ml^−1^ to 1.5 × 10^6^ crystals ml^−1^ yielded reasonable chip-loading levels that allowed us to collect sufficient data for a complete dataset with good signal-to-noise and merging statistics in an hour (*i.e.* two chips) (Schulz *et al.*, 2018 ▸; Mehrabi, Schulz, Dsouza *et al.*, 2019 ▸; Mehrabi, Schulz, Agthe *et al.*, 2019 ▸). Higher crystal concentrations result in higher loading levels, but also in a much higher percentage of multiple lattices (*i.e.* two or more crystals per well). This is problematic for TR-SSX as it makes the degree of reaction initiation by either light or on-chip mixing harder to assess.

#### 3.5.2. Chip loading with microcrystals   

Although the rules of thumb stated above are, in our experience, a good starting point, optimal crystal concentrations for each specific system should be determined by comparing the crystal concentration with the diffraction hit rate. This allows the suitable vacuum level during chip loading to be determined. For our previously used systems (lysozyme, xylose isomerase, fluoro­acetate dehalogenase), vacuum levels between 0.3 and 0.5 bar below atmospheric pressure resulted in efficient loading with no apparent crystal damage, while stronger vacuum levels resulted in visible damage to the crystals and in split diffraction spots (Schulz *et al.*, 2018 ▸; Mehrabi, Schulz, Dsouza *et al.*, 2019 ▸; Mehrabi, Schulz, Agthe *et al.*, 2019 ▸). However, these crystal systems are rather robust and more sensitive crystals of, for example, large macromolecular assemblies require much lower values than 0.3 bar, and these must be defined on a case-by-case basis. Importantly, regardless of the vacuum pressure used, to prevent dehydration of the crystals, suction should only be applied until the moment the crystallization mother liquor is removed from the chip surface. In summary, the crystal concentration determines how well a particular chip is loaded. Based on the sample type (crystal concentration, viscosity of the medium, fragility of the crystals, *etc*.) the vacuum level dictates how well the samples drain into the crystal wells. If the vacuum level is set too high, the crystals will break or dehydrate and thereby reduce the diffraction hit rate. Therefore, the chip-loading device allows careful optimization of loading parameters for each system, enabling effective loading and preventing dehydration and damage of crystals (Mehrabi, Schulz, Agthe *et al.*, 2019 ▸).

## 4. Discussion   

In comparison with our previous design, both the Oxford photochip and the HARE chip represent a substantial advance over the previous version (Zarrine-Afsar *et al.*, 2012 ▸; Oghbaey *et al.*, 2016 ▸; Ebrahim, Appleby *et al.*, 2019 ▸). With only a slight increase in the outer dimension the crystal density has been increased approximately twofold (Mueller *et al.*, 2015 ▸; Oghbaey *et al.*, 2016 ▸; Ebrahim, Appleby *et al.*, 2019 ▸). This has the clear advantage of increased data rates, as the most time-consuming step during data collection is the sample exchange and the re-alignment of the chip in the beam path. Importantly, since both chip designs share the same 30 × 30 mm form factor, all instrumentation and protocols described here are common to both chips. Other silicon chip designs with different form factors and much higher hole densities than the HARE or Oxford photochip have also been realized. These have been used for high-throughput data collections of static structures at cryogenic and room-temperature alike (Lieske *et al.*, 2019 ▸). However, the HARE chip has been specifically designed for time-resolved experiments.

In an optical pump–probe experiment, a laser pulse is used for reaction initiation. For signal optimization the pumped volume needs to exceed the probed volume by a factor of two to five. To achieve a homogenous sample excitation of a 5–10 µm probe area (X-ray microfocus beam) a UV laser pulse has to have a 50 µm diameter, assuming optimal beam alignment and a typical Gaussian profile. In practice, laser beam diameters are determined by the optics used and wavelength, but typically lie between 50 µm and 100 µm (FWHM) (Epp *et al.*, 2017 ▸; Schulz *et al.*, 2018 ▸). Thus, a pitch of the crystal wells smaller than 100–120 µm has the potential to lead to excitation of neighbouring crystals in high-density grids. However, a large pitch (distance) between crystal wells is not only required for optical reaction initiation. To circumvent the need for optical excitation we have recently extended the repertoire of possible reaction initiations to an on-chip mixing approach called LAMA. In the LAMA approach, droplets of soluble substrates are directly applied to each well of the chip (Mehrabi, Schulz, Agthe *et al.*, 2019 ▸). To prevent leaking of solution into neighbouring features, the pitch of the crystal wells has to be on the same scale as is required for optical pump–probe experiments. In the Oxford and HARE chips, leakage is further prevented by the pyramidal shape of the crystal wells. In contrast to flat-surface chips the pyramidal shaped crystal wells trap the applied droplet and guide it towards the crystals. Additionally, the well shape probably prevents preferred orientation problems of the crystals (Davy *et al.*, 2019 ▸). The larger well-to-well pitch and the shape of the wells make the Oxford and HARE chips extremely well suited to TR-SSX applications using both laser light and on-chip mixing approaches.

**Figure 9:**
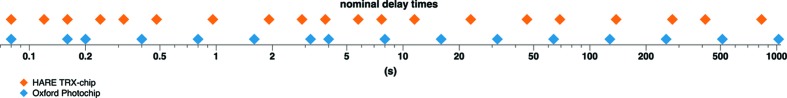
HARE delay times that can be addressed with the HARE chip and the Oxford photochip. The figure illustrates the discrete time points that can be addressed with the Oxford photochip (blue) and the HARE chip (orange) without changing any experimental parameter other than the time delay between reaction initiation pump and probe. Time delays are calculated for a translation-stage frequency of 25 Hz.

While both chip designs are suitable for time-resolved measurements, the advantage of the Oxford photochip is that it can accommodate a higher number of crystals (>25 000), enabling a greater amount of data to be collected per chip (Ebrahim, Appleby *et al.*, 2019 ▸). In contrast, the HARE chips with their high composite number of crystal wells per compartment are designed to make best use of the HARE algorithm for efficient time-resolved crystallographic data collection (Schulz *et al.*, 2018 ▸). This increases flexibility with respect to the resolvable time points without requiring more complicated rastering patterns during data collection. This directly addresses our aim of broadening the accessibility of time-resolved experiments, as it allows a user to conveniently resolve a large number of reaction intermediates without encountering experimental complications in designing the data-collection protocol. These practical implications become more apparent if one compares the time points that can be addressed for a whole chip (Fig. 9 ▸). Usually the *in crystallo* kinetics of an enzymatic reaction are unknown before conducting a time-resolved experiment. Under these circumstances it is of great advantage to initially characterize the reaction intermediates on a logarithmic timescale, which provides a systematic overview with the least number of snapshots along the reaction coordinate. On a logarithmic scale the delay times that can be addressed by the HARE chip are distributed more linearly, in particular up to 10 s of delay time, which encompasses the majority of enzymatic turnovers (Bar-Even *et al.*, 2011 ▸). Once the critical time points have been identified they can of course be re-addressed with a more fine-sliced data-collection strategy.

Obviously, it would be desirable to further increase the number of crystal wells per compartment to address a much larger number of discrete time points. However, using prototypes with up to 53 × 53 crystal wells per compartment we found that the fragility of the compartment areas drastically increases. This prevents the compartments from withstanding even small vacuum levels and results in chips breaking during crystal loading. Different material properties and/or different aspect ratios of the compartment areas might mitigate this effect and it is therefore possible that higher HARE numbers can be realized with different chip designs or materials. Currently, however, the 24 × 24 crystal well per compartment design appears to be a sweet spot between chip resilience and time-point versatility. Notably, with a change in the translation frequency the HARE delay times change accordingly, providing access to arbitrary delay times for all chip types.

In summary, in order to match the HARE chip with a versatile loading solution we have developed a cost-effective modular chip-loading platform that can be easily implemented at any beamline. To address the requirement to load un­damaged crystals, under controlled humidity and for a wide variety of buffer systems a vacuum-based solution seems to provide the most versatile chip-loading approach. We can limit physical stress by adapting vacuum levels and evacuation time to both crystals and buffer system and conveniently control the humidity in the chip-loading environment to prevent dehydration of the crystals during the loading process.

By providing here all the required information to reproduce the tools and protocols we have described here, we hope to aid beamline staff and users in establishing TR-SSX at other synchrotron sources. Clearly, a larger community engaged in such studies will aid in further refining protocols to reduce crystal consumption and to make TR-SSX a routine method at synchrotrons that is easily accessible to the large number of non-expert users with burning biological questions that can be addressed by time-resolved structural studies.

## Supplementary Material

Figure S1: SEM Images of the HARE chip at different magnifications . DOI: 10.1107/S1600577520000685/wz5004sup1.pdf

Supporting material 1 to 10 (CAD, 3D printer files, etc.) to reproduce the HARE chip and the accessory material. . DOI: 10.1107/S1600577520000685/wz5004sup2.zip
