# Supplementary material for: The HARE chip for efficient time-resolved serial synchrotron crystallography
Source: J Synchrotron Radiat. 2020 Feb 27;27(Pt 2):360–70. doi: 10.1107/S1600577520000685 (PMC7064102; doi:10.1107/S1600577520000685)

JOURNAL OF
SYNCHROTRON
RADIATION

Volume 27 (2020)

Supporting information for article:

The HARE chip for efficient time-resolved serial synchrotron crystallography

Pedram Mehrabi, Henrike Müller-Werkmeister, Jan-Philipp Leimkohl, Hendrik Schikora, Jelena Ninkovic, Silvia Krivokuca, Ladislav Andriček, Sascha W. Epp, Darren Sherrel, Robin L. Owen, Arwen R. Pearson, Friedjof Tellkamp, Eike C. Schulz and R. J. Dwayne Miller

Figure S1 SEM images of the HARE chip at different magnifications.

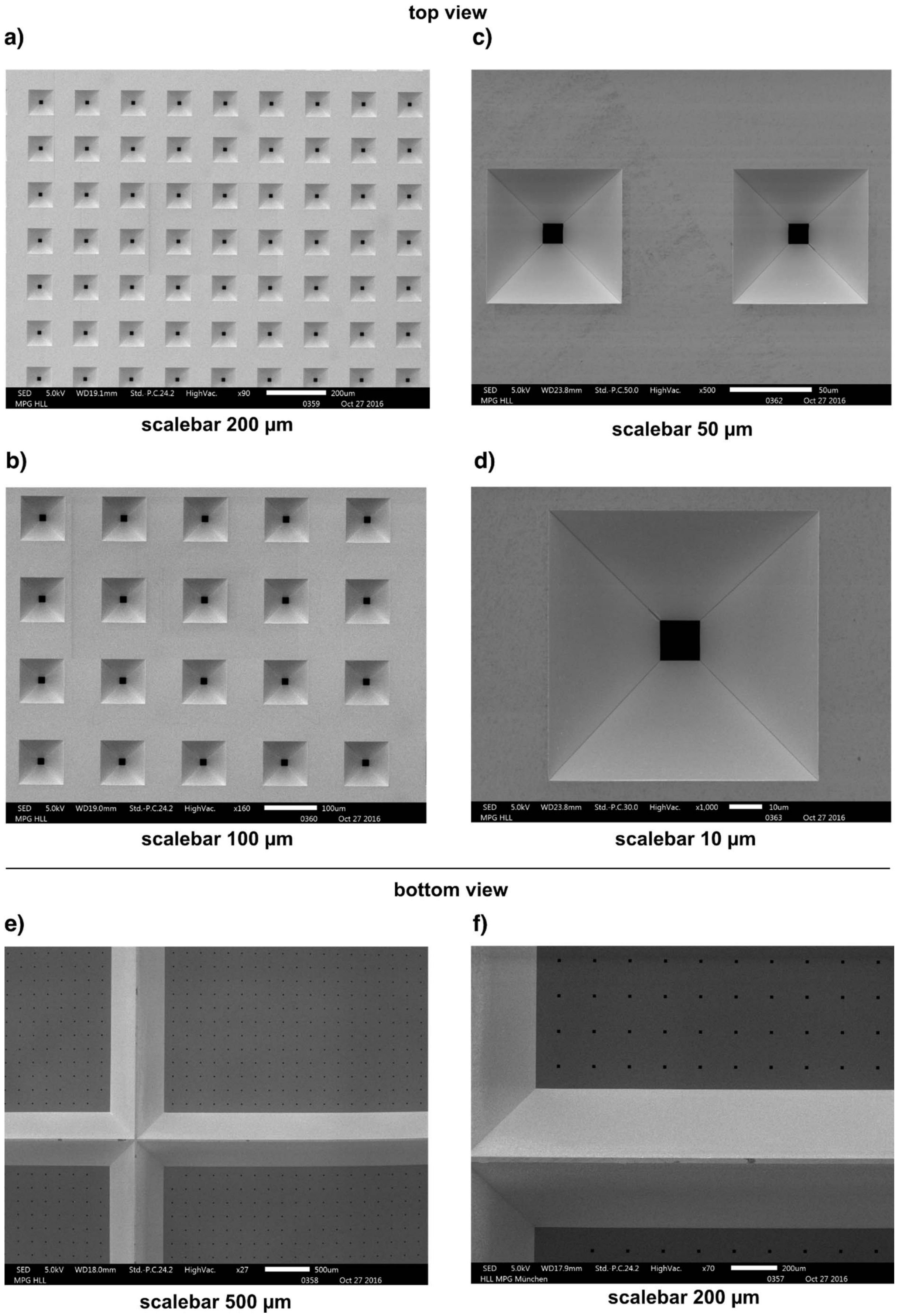

Supplement: Supplementary file 1 [file s-27-00360-sup1.pdf]
