# Supplementary material for: The HARE chip for efficient time-resolved serial synchrotron crystallography
Source: J Synchrotron Radiat. 2020 Feb 27;27(Pt 2):360–70. doi: 10.1107/S1600577520000685 (PMC7064102; doi:10.1107/S1600577520000685)
Supplement: Supplementary file 2 [file s-27-00360-sup2.zip › 01_SupMat1_chip/HLL 6x6x24x24.pdf]

\\win.desy.de\group\cfel\4all\mpsd_drive\SSU-MP\00 SSU projects\SSU-MP0017 EMBL P14\4 Mechanics\20191101 SE chips\HLL 6x6x24x24.dft

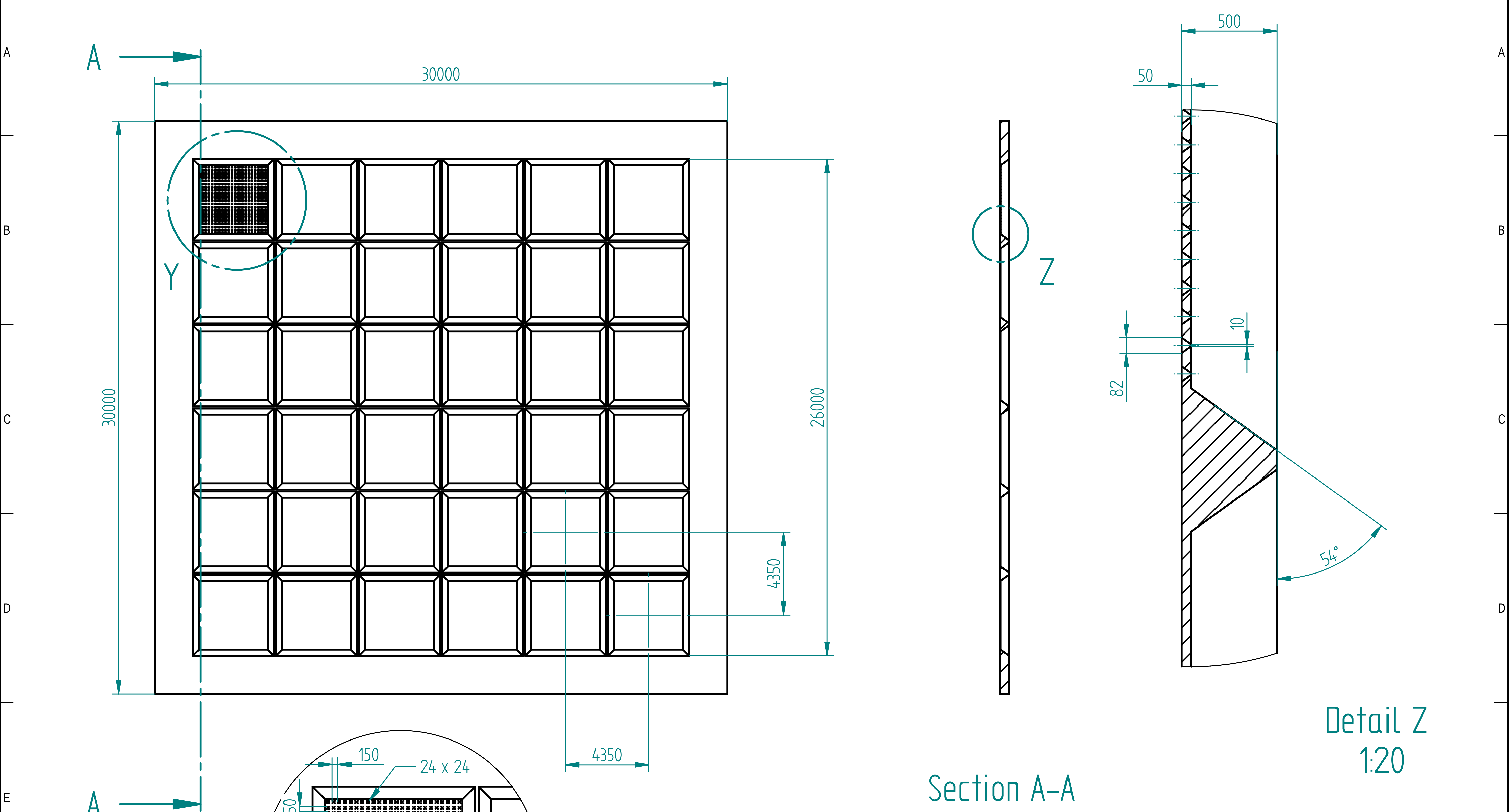

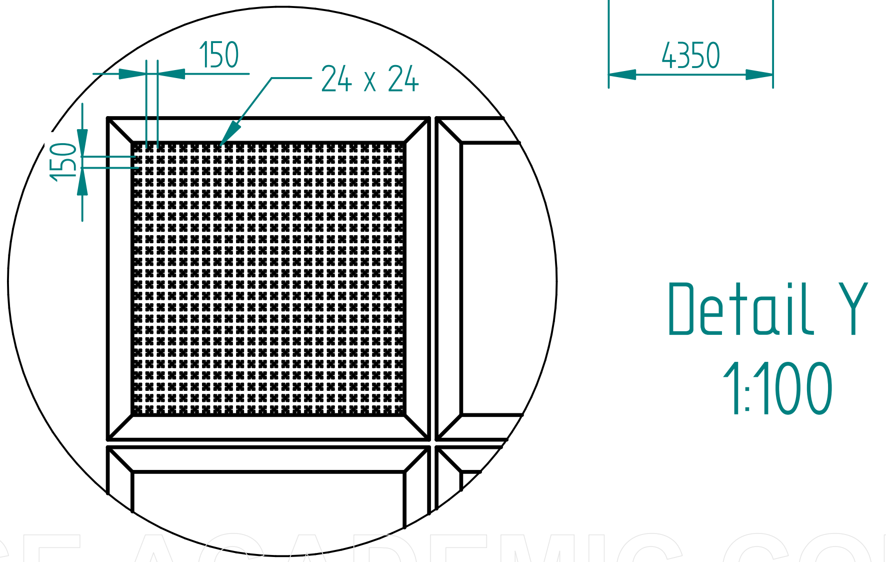

Toleranz - klasse	Allgemeintoleranzen für Rechtwinkligkeit in mm bis 100	über 100 bis 300	über 300 bis 1000	über 1000 bis 3000
H	0,2	0,3	0,4	0,5
K	0,4	0,6	0,8	1
L	0,6	1	1,5	2

Toleranz - klasse	Allgemeintoleranzen für Geradheit und Ebenheit in mm bis 10	über 10 bis 30	über 30 bis 100	über 100 bis 300	über 300 bis 1000	über 1000 bis 3000
H	0,02	0,05	0,1	0,2	0,3	0,4
K	0,05	0,1	0,2	0,4	0,6	0,8
L	0,1	0,2	0,4	0,8	1,2	1,6

Toleranz - klasse	Grenzabmaße in mm für Nennmaßbereich in mm (ISO 2768) bis 0,5	über 0,5 bis 3	über 3 bis 6	über 6 bis 30	über 30 bis 120	über 120 bis 400	über 400 bis 1000	über 1000 bis 2000	über 2000 bis 4000	über 4000 bis 8000
f (fein)	direkt am	± 0,05	± 0,05	± 0,10	± 0,15	± 0,2	± 0,3	± 0,5	-	-
m (mittel)	Nennmaß	± 0,10	± 0,10	± 0,20	± 0,30	± 0,5	± 0,8	± 1,2	± 2	± 3
g (grob)	angeben	± 0,15	± 0,20	± 0,50	± 0,80	± 1,2	± 2,0	± 3,0	± 4	± 5

SOLID EDGE ACADEMIC COPY

K-Zchng.-ID C-DRAW.-ID		K-Rev. C-REV.	K-Status K-STATUS 0–Verfügbar	
Projekt / PROJECT	Arbeitspaket / WORKPACKAGE	Gruppe / GROUP MPSD–AR	Ers.für / REPLACES	Ers.durch / REPLACED BY
Gewicht / WEIGHT Fehler: Keine Referenz	Halbzeug / SEMIFINISHED PRODUCT	mpsd CFEL	Werkstoff / MATERIAL Silicon 100	Format/SIZE
Allg.Toleranzen / GENERAL TOLERANCES ISO 2768 ISO 13920; Tolerierungsgrundsatz / FUNDAMENTAL TOLERANCING PRINCIPLE ISO 8015; Oberflächenkenngrößen / SURFACE TEXTURE ISO 1302 4287, 4288	Toleranzklasse / TOLERANCE CLASS	Maßstab / SCALE Maßstab; Teile-ID PART-ID	Titel / TITLE HLL 6x6x24x24	

	Datum / DATE	Name / NAME
Gez. CRE.	04.11.19	tellkamf
Gen. APR.		
Frei. REL.		
Gepr. REV.		

Dokument-Nr. / DOCUMENT NO.

Blatt SHEET 1 von OF 1

Zchng.-ID DRAW.-ID | Rev. REV. | Ver. VER. | Status STATUS

© CFEL-MPSD behält sich alle Rechte vor. Schutzvermerk ISO 16016 beachten.

©MPSD. ALL RIGHTS RESERVED. PREFERRED TO PROTECTION NOTICE ISO 16016.
