# Supplementary material for: The HARE chip for efficient time-resolved serial synchrotron crystallography
Source: J Synchrotron Radiat. 2020 Feb 27;27(Pt 2):360–70. doi: 10.1107/S1600577520000685 (PMC7064102; doi:10.1107/S1600577520000685)
Supplement: Supplementary file 2 [file s-27-00360-sup2.zip › 07_SupMAt7_loading device/upper clamp.pdf]

M:\00 SSU projects\SSU-MP0128 Suction Device\4 Mechanics\upper clamp.dft

Allgemeintoleranzen für Rechtwinkligkeit in mm				
Toleranz - klasse	bis 100	über 100 bis 300	über 300 bis 1000	über 1000 bis 3000
H	0,2	0,3	0,4	0,5
K	0,4	0,6	0,8	1
L	0,6	1	1,5	2

Allgemeintoleranzen für Geradheit und Ebenheit in mm						
Toleranz - klasse	bis 10	über 10 bis 30	über 30 bis 100	über 100 bis 300	über 300 bis 1000	über 1000 bis 3000
H	0,02	0,05	0,1	0,2	0,3	0,4
K	0,05	0,1	0,2	0,4	0,6	0,8
L	0,1	0,2	0,4	0,8	1,2	1,6

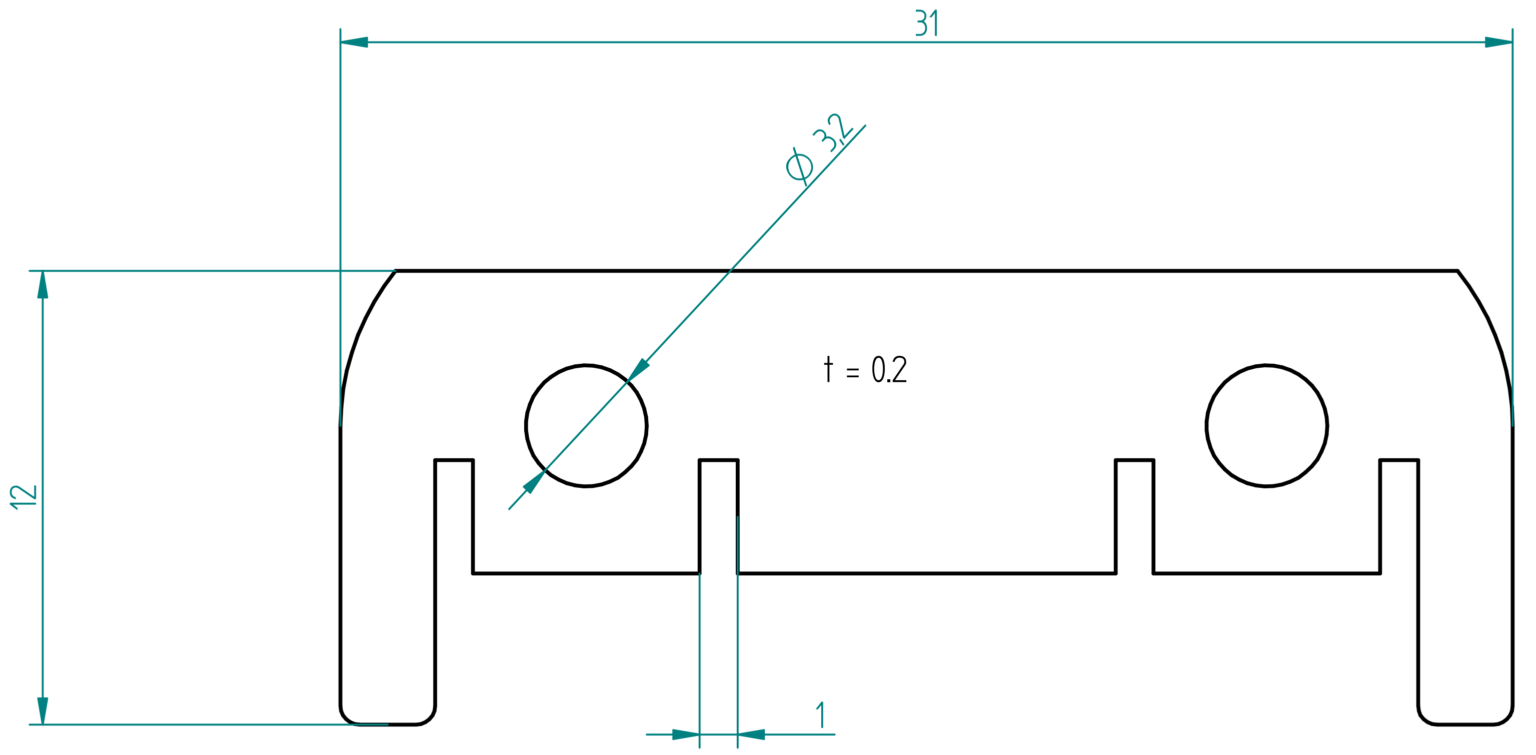

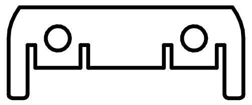

1:1

Edelstahl
2 Steuck

Ra 3,2

-0,1
-0,3

+0,3
+0,1

Grenzabmaße in mm für Nennmaßbereich in mm (ISO 2768)										
Toleranz - klasse	bis 0,5	über 0,5 bis 3	über 3 bis 6	über 6 bis 30	über 30 bis 120	über 120 bis 400	über 400 bis 1000	über 1000 bis 2000	über 2000 bis 4000	über 4000 bis 8000
f (fein)	direkt am	± 0,05	± 0,05	± 0,10	± 0,15	± 0,2	± 0,3	± 0,5	-	-
m (mittel)	Nennmaß	± 0,10	± 0,10	± 0,20	± 0,30	± 0,5	± 0,8	± 1,2	± 2	± 3
g (grob)	angeben	± 0,15	± 0,20	± 0,50	± 0,80	± 1,2	± 2,0	± 3,0	± 4	± 5

SOLID EDGE ACADEMIC COPY

K-Zchng.-ID C-DRAW.-ID | K-Rev. C-REV. | K-Status K-STATUS 0-Verfügbar

Projekt / PROJECT

Arbeitspaket / WORKPACKAGE

Gruppe / GROUP: MPSD-AR

Ers.für / REPLACES

Ers.durch / REPLACED BY

Gewicht / WEIGHT: Fehler: Keine Referenz

Halbzeug / SEMIFINISHED PRODUCT

mpsd CFEL

Werkstoff / MATERIAL

Format/SIZE

Allg.Toleranzen / GENERAL TOLERANCES ISO 2768 ISO 13920

Tolerierungsgrundsatz / FUNDAMENTAL TOLERANCING PRINCIPLE ISO 8015

Oberflächenkenngrößen / SURFACE TEXTURE ISO 1302 4287, 4288

Toleranzklasse / TOLERANCE CLASS: fH

Maßstab / SCALE: Maßstab

Teile-ID PART-ID

Titel / TITLE

	Datum / DATE	Name / NAME
Gez. CRE.	25.03.19	tellkamf
Gen. APR.		
Frei. REL.		
Gepr. REV.		

© CFEL-MPSD behält sich alle Rechte vor. Schutzvermerk ISO 16016 beachten.

©MPSD. ALL RIGHTS RESERVED. PREFERRED TO PROTECTION NOTICE ISO 16016.

Dokument-Nr. / DOCUMENT NO.

Blatt SHEET 1 von OF 1

Zchng.-ID DRAW.-ID | Rev. REV. | Ver. VER. | Status STATUS
