# Supplementary material for: The HARE chip for efficient time-resolved serial synchrotron crystallography
Source: J Synchrotron Radiat. 2020 Feb 27;27(Pt 2):360–70. doi: 10.1107/S1600577520000685 (PMC7064102; doi:10.1107/S1600577520000685)
Supplement: Supplementary file 2 [file s-27-00360-sup2.zip › 10_SupMat10_humidityHood/001_Glovebox-SE000864567.pdf]

K:\001_PROJEKTE_mpsd_#\Projekte_2015\15-0072_Glovebox\001_Glovebox-SE000864567.dft

| Allgemeintoleranzen für Rechtwinkligkeit in mm | | | | |
|---|---|---|---|---|
| Toleranz - klasse | bis 100 | über 100 bis 300 | über 300 bis 1000 | über 1000 bis 3000 |
| H | 0,2 | 0,3 | 0,4 | 0,5 |
| K | 0,4 | 0,6 | 0,8 | 1 |
| L | 0,6 | 1 | 1,5 | 2 |

| Allgemeintoleranzen für Geradheit und Ebenheit in mm | | | | | | |
|---|---|---|---|---|---|---|
| Toleranz - klasse | bis 10 | über 10 bis 30 | über 30 bis 100 | über 100 bis 300 | über 300 bis 1000 | über 1000 bis 3000 |
| H | 0,02 | 0,05 | 0,1 | 0,2 | 0,3 | 0,4 |
| K | 0,05 | 0,1 | 0,2 | 0,4 | 0,6 | 0,8 |
| L | 0,1 | 0,2 | 0,4 | 0,8 | 1,2 | 1,6 |

| Toleranz - klasse | Grenzabmaße in mm für Nennmaßbereich in mm ( ISO 2768 ) | | | | | | | | | |
|---|---|---|---|---|---|---|---|---|---|---|
| | bis 0,5 | über 0,5 bis 3 | über 3 bis 6 | über 6 bis 30 | über 30 bis 120 | über 120 bis 400 | über 400 bis 1000 | über 1000 bis 2000 | über 2000 bis 4000 | über 4000 bis 8000 |
| f (fein) | direkt am | ± 0,05 | ± 0,05 | ± 0,10 | ± 0,15 | ± 0,2 | ± 0,3 | ± 0,5 | - | - |
| m (mittel) | Nennmaß | ± 0,10 | ± 0,10 | ± 0,20 | ± 0,30 | ± 0,5 | ± 0,8 | ± 1,2 | ± 2 | ± 3 |
| g (grob ) | angeben | ± 0,15 | ± 0,20 | ± 0,50 | ± 0,80 | ± 1,2 | ± 2,0 | ± 3,0 | ± 4 | ± 5 |

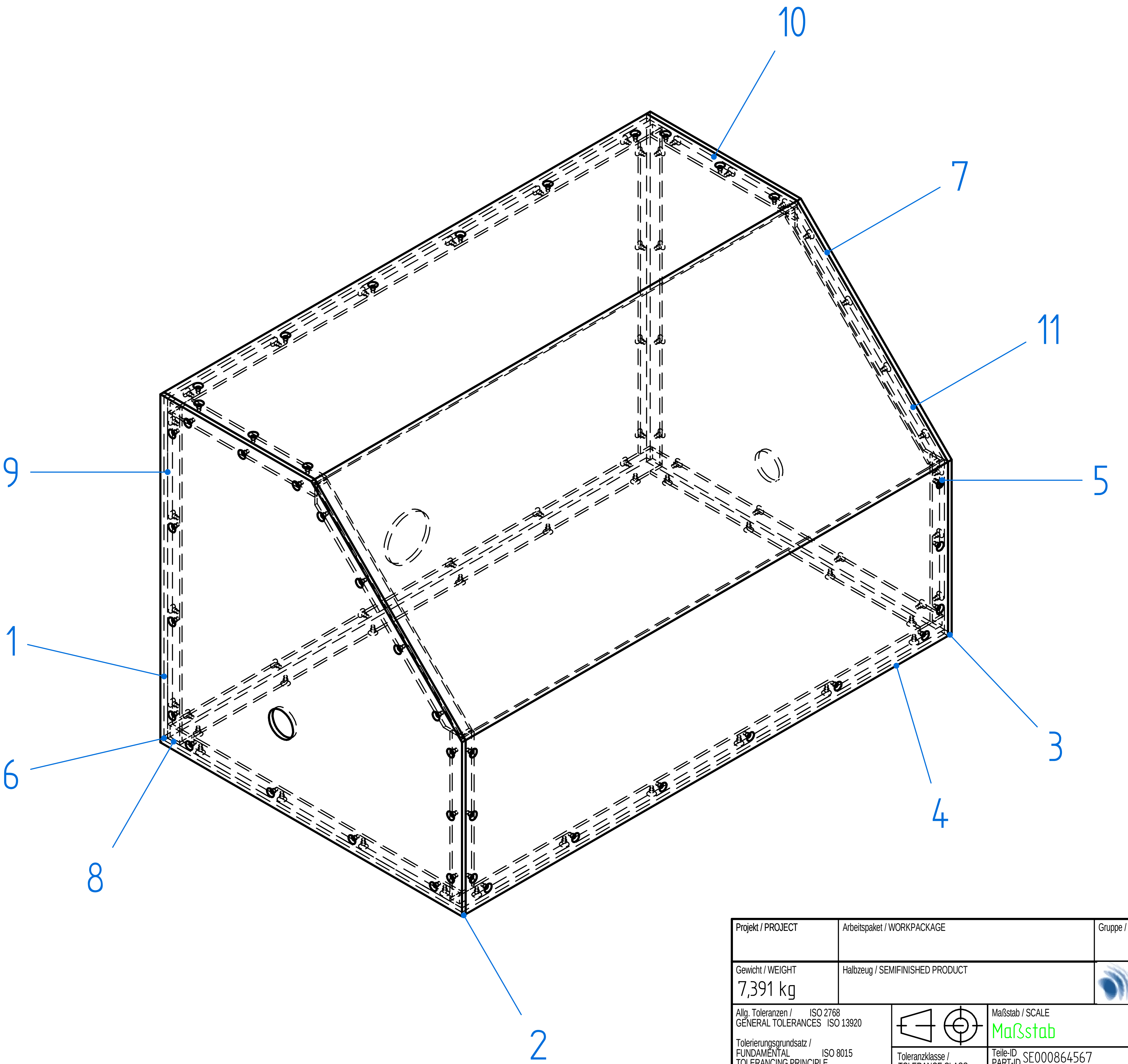


**Nur zur Information**

Nicht nach dieser Zeichnung fertigen!

| | | |
|---|---|---|
| K-Zchng.-ID C-DRAW.-ID | K-Rev. C-REV. | K-Status K-STATUS 0-Verfügbar |
| Ers.für / REPLACES | Ers.durch / REPLACED BY | |

Projekt / PROJECT

Arbeitspaket / WORKPACKAGE

Gruppe / GROUP

Gewicht / WEIGHT: 7,391 kg

Halbzeug / SEMIFINISHED PRODUCT

mpsd CFEL

Werkstoff / MATERIAL

Format/SIZE

Allg. Toleranzen / GENERAL TOLERANCES ISO 2768 ISO 13920

Tolerierungsgrundsatz / FUNDAMENTAL TOLERANCING PRINCIPLE ISO 8015

Oberflächenkenngrößen / SURFACE TEXTURE ISO 1302 4287, 4288

Toleranzklasse / TOLERANCE CLASS

Maßstab / SCALE: Maßstab

Teile-ID PART-ID: SE000864567

Titel / TITLE: **Glovebox**

| | Datum / DATE | Name / NAME |
|---|---|---|
| Gez. CRE. | 19.06.17 | tellkamf |
| Gen. APR. | | |
| Frei. REL. | | |
| Gepr. REV. | | |

Dokument-Nr. / DOCUMENT NO.: 15-0072-0-000

Blatt SHEET 1 von OF 2

Zchng.-ID DRAW.-ID | Rev. REV. | Ver. VER. | Status STATUS

© CFEL-MPSD behält sich alle Rechte vor. Schutzvermerk ISO 16016 beachten. Für Rückfragen bitte an -TT- wenden

©MPSD. ALL RIGHTS RESERVED. PREFERRED TO PROTECTION NOTICE ISO 16016. FOR FURTHER ENQUIRIES PLEASE CONTACT -TT-

K:\001_PROJEKTE_mpsd_#\Projekte_2015\15-0072_Glovebox\001_Glovebox-SE000864567.dft

| Pos | Titel | DokumentNr.-DIN/ISO | Stk. | Werkstoff | Untertitel | Halbzeug/Bemerkungen | Teilenummer |
|---|---|---|---|---|---|---|---|
| 1 | Backplatte | 15-0072-0-002 | 1 | PMMA | | | SE000864569 |
| 2 | Seitenplatte_2 | 15-0072-0-00xx | 1 | PMMA | | | SE000864577 |
| 3 | Seitenplatte_2 | 15-0072-0-00xx | 1 | PMMA | | | SE000864577 |
| 4 | Platte -oben | 15-0072-0-001 | 2 | PMMA | | | SE000864570 |
| 5 | Senkschraube ISO 10642 M5x20 | | 1 | A4 | | | SE000773920 |
| 6 | Bodenplatte | 15-0072-0-00xx | 1 | PMMA | | | SE000864585 |
| 7 | Platte -oben | 15-0072-0-001 | 1 | PMMA | | | SE000864570 |
| 8 | Winkel 20x20x4 lang | 15-0072-0-00xx | 3 | Al | | | SE000864874 |
| 9 | Winkel 20x20x4 2 | 15-0072-0-00xx | 4 | Al | | | SE000864951 |
| 10 | Winkel 20x20x4 3 | 15-0072-0-00xx | 4 | Al | | | SE000864986 |
| 11 | Winkel 20x20x4 4 | 15-0072-0-00xx | 2 | Al | | | SE000864987 |

Nur zur Information

Nicht nach dieser Zeichnung fertigen!

Pos.-Nr. mit * sind auf der Zeichnung nicht angezogen

K-Zchng.-ID / C-DRAW.-ID

K-Rev. /C-REV .

K-Status / C-STATUS: 0-Verfügbar

Gruppe / GROUP

mpsd CFEL

Arbeitspaket / WORKPACKAGE

Ers.für / REPLACES

Projekt / PROJECT

Ers.durch / REPLACED BY

Allg. Toleranzen / GENERAL TOLREANCES: ISO 2768, ISO 13920

Tolerierungsgrundsatz / FUNDAMENTAL TOLERANCING PRINCIPLE: ISO 8015

Oberflächenkenngrößen / SURFACE TEXTURE: ISO 1302, 4287, 4288

Toleranzklasse / TOLERANCE CLASS

© CFEL-MPSD behält sich alle Rechte vor. Schutzvermerk ISO 16016 beachten. Für Rückfragen bitte an -TT- wenden

©MPSD. ALL RIGHTS RESERVED. PREFERRED TO PROTECTION NOTICE ISO 16016. FOR FURTHER ENQUIRIES PLEASE CONTACT -TT-

Maßstab / SCALE: Maßstab

Teile-ID / PART-ID: SE000864567

| | Datum/DATE | Name / NAME |
|---|---|---|
| Gez. CRE. | 19.06.17 | tellkamf |
| Gepr. REV. | | |
| Frei. REL. | | |
| Gen. APR. | | |

Titel / TITLE

# Glovebox

Dokument-Nr. / DOCUMENT NO.: 15-0072-0-000

Blatt SHEET 2 von OF 2

Zchng.-ID DRAW.-ID | Rev. REV. | Ver. VER. | Status STATUS
