# Supplementary material for: The HARE chip for efficient time-resolved serial synchrotron crystallography
Source: J Synchrotron Radiat. 2020 Feb 27;27(Pt 2):360–70. doi: 10.1107/S1600577520000685 (PMC7064102; doi:10.1107/S1600577520000685)
Supplement: Supplementary file 2 [file s-27-00360-sup2.zip › 10_SupMat10_humidityHood/15-0072-0-001_Backplatte-SE000869530.pdf]

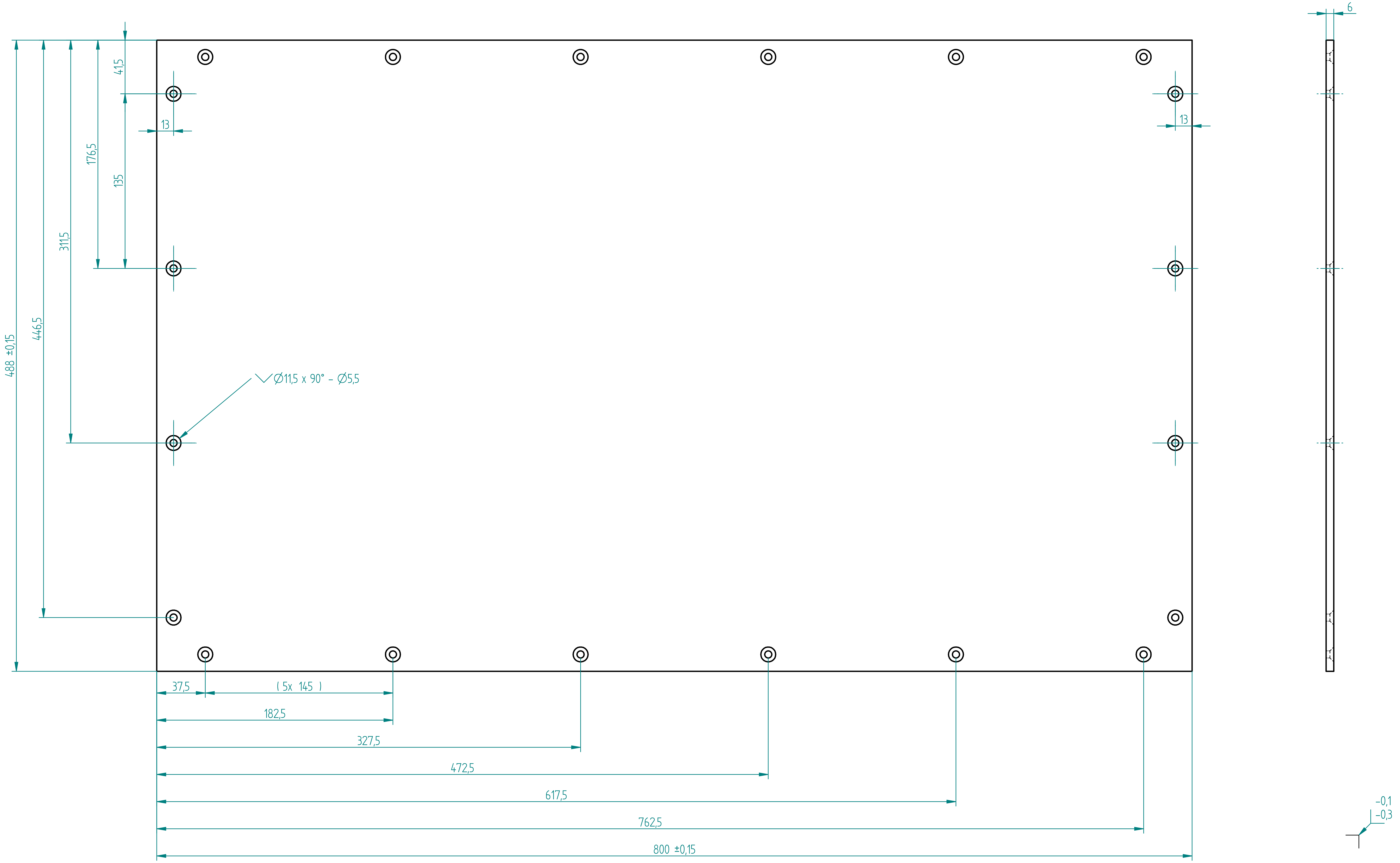

K-Zchng.-ID / C-DRAW.-ID	SE000850350	K-Rev. / C-REV.		K-Status / K-STATUS	0-Verfügbar
Projekt / PROJECT	Arbeitspaket / WORKPACKAGE	Gruppe / GROUP	Ers.für / REPLACES	Ers.durch / REPLACED BY	
Gewicht / WEIGHT: 2,731 kg	Halbzeug / SEMIFINISHED PRODUCT	mpsd CFEL	Werkstoff / MATERIAL: PMMA (Plexiglas)	Format/SIZE	

Allg. Toleranzen / GENERAL TOLERANCES ISO 2768, ISO 13920
Tolerierungsgrundsatz / FUNDAMENTAL TOLERANCING PRINCIPLE ISO 8015
Oberflächenkenngrößen / SURFACE TEXTURE ISO 1302, 4287, 4288

Toleranzklasse / TOLERANCE CLASS: fH

Maßstab / SCALE: 1 : 2

Teile-ID / PART-ID: SE000864569

Titel / TITLE: Backplatte

	Datum / DATE	Name / NAME
Gez. CRE.	08.09.16	gonschj
Gen. APR.		
Frei. REL.		
Gepr. REV.		

© CFEL-MPSD behält sich alle Rechte vor. Schutzvermerk ISO 16016 beachten. Für Rückfragen bitte an -TT- wenden

©MPSD. ALL RIGHTS RESERVED. PREFERRED TO PROTECTION NOTICE ISO 16016. FOR FURTHER ENQUIRIES PLEASE CONTACT -TT-

Dokument-Nr. / DOCUMENT NO.: 15-0072-0-002

Blatt / SHEET 1 von / OF 1

Zchng.-ID / DRAW.-ID | Rev. / REV. | Ver. / VER. | Status / STATUS

K:\001_PROJEKTE_mpsd_#\Projekte_2015\15-0072_Glovebox\15-0072-0-001_Backplatte-SE000869530.dft
