# Supplementary material for: The HARE chip for efficient time-resolved serial synchrotron crystallography
Source: J Synchrotron Radiat. 2020 Feb 27;27(Pt 2):360–70. doi: 10.1107/S1600577520000685 (PMC7064102; doi:10.1107/S1600577520000685)
Supplement: Supplementary file 2 [file s-27-00360-sup2.zip › 10_SupMat10_humidityHood/15-0072-0-00x_Winkel_20x20__3-SE000869516.pdf]

K:\001_PROJEKTE_mpsd_#\Projekte_2015\15-0072_Glovebox\15-0072-0-00x_Winkel_20x20__3-SE000869516.dft

Allgemeintoleranzen für Rechtwinkligkeit in mm				
Toleranz-klasse	bis 100	über 100 bis 300	über 300 bis 1000	über 1000 bis 3000
H	0,2	0,3	0,4	0,5
K	0,4	0,6	0,8	1
L	0,6	1	1,5	2

Allgemeintoleranzen für Geradheit und Ebenheit in mm						
Toleranz-klasse	bis 10	über 10 bis 30	über 30 bis 100	über 100 bis 300	über 300 bis 1000	über 1000 bis 3000
H	0,02	0,05	0,1	0,2	0,3	0,4
K	0,05	0,1	0,2	0,4	0,6	0,8
L	0,1	0,2	0,4	0,8	1,2	1,6

Grenzabmaße in mm für Nennmaßbereich in mm (ISO 2768)										
Toleranz-klasse	bis 0,5	über 0,5 bis 3	über 3 bis 6	über 6 bis 30	über 30 bis 120	über 120 bis 400	über 400 bis 1000	über 1000 bis 2000	über 2000 bis 4000	über 4000 bis 8000
f (fein)	direkt am	± 0,05	± 0,05	± 0,10	± 0,15	± 0,2	± 0,3	± 0,5	-	-
m (mittel)	Nennmaß	± 0,10	± 0,10	± 0,20	± 0,30	± 0,5	± 0,8	± 1,2	± 2	± 3
g (grob)	angeben	± 0,15	± 0,20	± 0,50	± 0,80	± 1,2	± 2,0	± 3,0	± 4	± 5

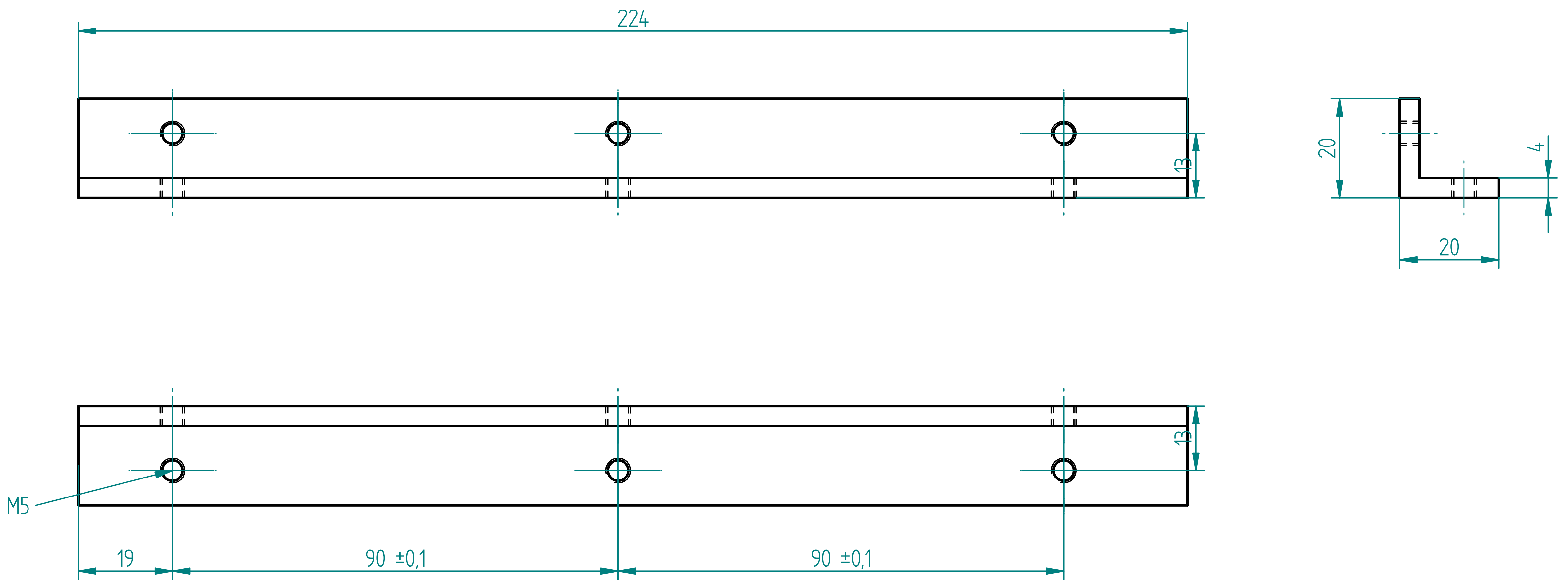

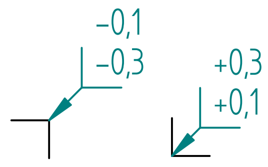

Kanten ISO 13715

K-Zchng.-ID C-DRAW.-ID SE000850345	K-Rev. C-REV.	K-Status K-STATUS 0-Verfügbar	
Projekt / PROJECT	Arbeitspaket / WORKPACKAGE	Gruppe / GROUP	Ers.für / REPLACES — Ers.durch / REPLACED BY
Gewicht / WEIGHT 0,086 kg	Halbzeug / SEMIFINISHED PRODUCT	mpsd CFEL	Werkstoff / MATERIAL Al — Format/SIZE
Allg. Toleranzen / GENERAL TOLERANCES ISO 2768 ISO 13920; Tolerierungsgrundsatz / FUNDAMENTAL TOLERANCING PRINCIPLE ISO 8015; Oberflächenkenngrößen / SURFACE TEXTURE ISO 1302 4287, 4288	Toleranzklasse / TOLERANCE CLASS fH	Maßstab / SCALE 1 : 1; Teile-ID PART-ID SE000864986	Titel / TITLE Winkel 20x20x4 3
		Datum / DATE — Name / NAME; Gez. CRE. 08.09.16 gonschj; Gen. APR.; Frei. REL.; Gepr. REV.	Dokument-Nr. / DOCUMENT NO. 15-0072-0-00xx; Blatt SHEET 1 von OF 1; Zchng.-ID DRAW.-ID; Rev. REV.; Ver. VER.; Status STATUS

© CFEL-MPSD behält sich alle Rechte vor. Schutzvermerk ISO 16016 beachten. Für Rückfragen bitte an -TT- wenden

©MPSD. ALL RIGHTS RESERVED. PREFERRED TO PROTECTION NOTICE ISO 16016. FOR FURTHER ENQUIRIES PLEASE CONTACT -TT-
