# Supplementary material for: The HARE chip for efficient time-resolved serial synchrotron crystallography
Source: J Synchrotron Radiat. 2020 Feb 27;27(Pt 2):360–70. doi: 10.1107/S1600577520000685 (PMC7064102; doi:10.1107/S1600577520000685)
Supplement: Supplementary file 2 [file s-27-00360-sup2.zip › 10_SupMat10_humidityHood/15-0072-0-00x_Winkel_20x20__4-SE000869517.pdf]

K:\001_PROJEKTE_mpsd_#\Projekte_2015\15-0072_Glovebox\15-0072-0-00x_Winkel_20x20__4-SE000869517.dft

Allgemeintoleranzen für Rechtwinkligkeit in mm				
Toleranz - klasse	bis 100	über 100 bis 300	über 300 bis 1000	über 1000 bis 3000
H	0,2	0,3	0,4	0,5
K	0,4	0,6	0,8	1
L	0,6	1	1,5	2

Allgemeintoleranzen für Geradheit und Ebenheit in mm						
Toleranz - klasse	bis 10	über 10 bis 30	über 30 bis 100	über 100 bis 300	über 300 bis 1000	über 1000 bis 3000
H	0,02	0,05	0,1	0,2	0,3	0,4
K	0,05	0,1	0,2	0,4	0,6	0,8
L	0,1	0,2	0,4	0,8	1,2	1,6

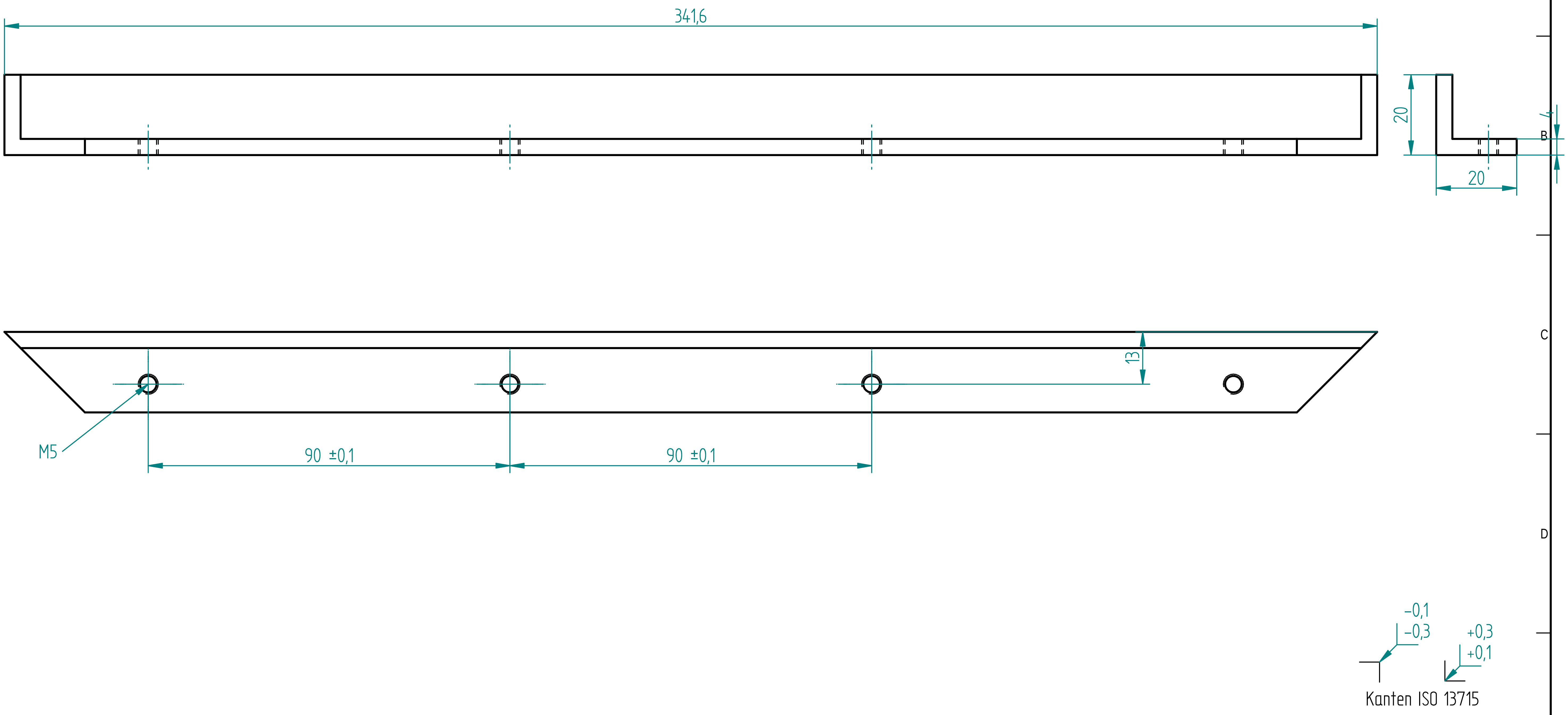

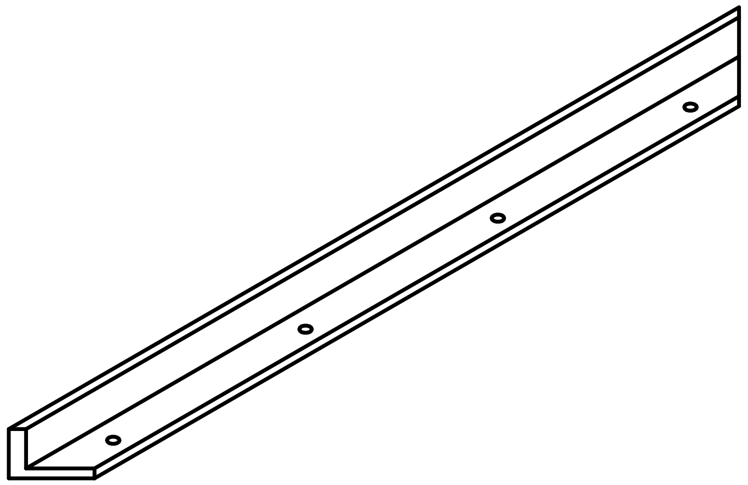

Grenzabmaße in mm für Nennmaßbereich in mm (ISO 2768)										
Toleranz - klasse	bis 0,5	über 0,5 bis 3	über 3 bis 6	über 6 bis 30	über 30 bis 120	über 120 bis 400	über 400 bis 1000	über 1000 bis 2000	über 2000 bis 4000	über 4000 bis 8000
f (fein)	direkt am	± 0,05	± 0,05	± 0,10	± 0,15	± 0,2	± 0,3	± 0,5	-	-
m (mittel)	Nennmaß	± 0,10	± 0,10	± 0,20	± 0,30	± 0,5	± 0,8	± 1,2	± 2	± 3
g (grob)	angeben	± 0,15	± 0,20	± 0,50	± 0,80	± 1,2	± 2,0	± 3,0	± 4	± 5

K-Zchng.-ID / C-DRAW.-ID: SE000850345
K-Rev. / C-REV.:
K-Status / K-STATUS: 0-Verfügbar

Projekt / PROJECT:
Arbeitspaket / WORKPACKAGE:
Gruppe / GROUP:
Ers.für / REPLACES:
Ers.durch / REPLACED BY:

Gewicht / WEIGHT: 0,127 kg
Halbzeug / SEMIFINISHED PRODUCT:
mpsd CFEL
Werkstoff / MATERIAL: Al
Format/SIZE:

Allg. Toleranzen / GENERAL TOLERANCES: ISO 2768, ISO 13920
Tolerierungsgrundsatz / FUNDAMENTAL TOLERANCING PRINCIPLE: ISO 8015
Oberflächenkenngrößen / SURFACE TEXTURE: ISO 1302, 4287, 4288
Toleranzklasse / TOLERANCE CLASS: fH

Maßstab / SCALE: 1 : 1
Teile-ID / PART-ID: SE000864987

Titel / TITLE: Winkel 20x20x4 4

	Datum / DATE	Name / NAME
Gez. / CRE.	08.09.16	gonschj
Gen. / APR.		
Frei. / REL.		
Gepr. / REV.		

© CFEL-MPSD behält sich alle Rechte vor. Schutzvermerk ISO 16016 beachten. Für Rückfragen bitte an -TT- wenden

©MPSD. ALL RIGHTS RESERVED. PREFERRED TO PROTECTION NOTICE ISO 16016. FOR FURTHER ENQUIRIES PLEASE CONTACT -TT-

Dokument-Nr. / DOCUMENT NO.: 15-0072-0-00xx
Blatt / SHEET: 1 von / OF: 1
Zchng.-ID / DRAW.-ID:
Rev. / REV.:
Ver. / VER.:
Status / STATUS:
