# Supplementary figures and images for: The HARE chip for efficient time-resolved serial synchrotron crystallography
Source: J Synchrotron Radiat. 2020 Feb 27;27(Pt 2):360–70. doi: 10.1107/S1600577520000685 (PMC7064102; doi:10.1107/S1600577520000685)

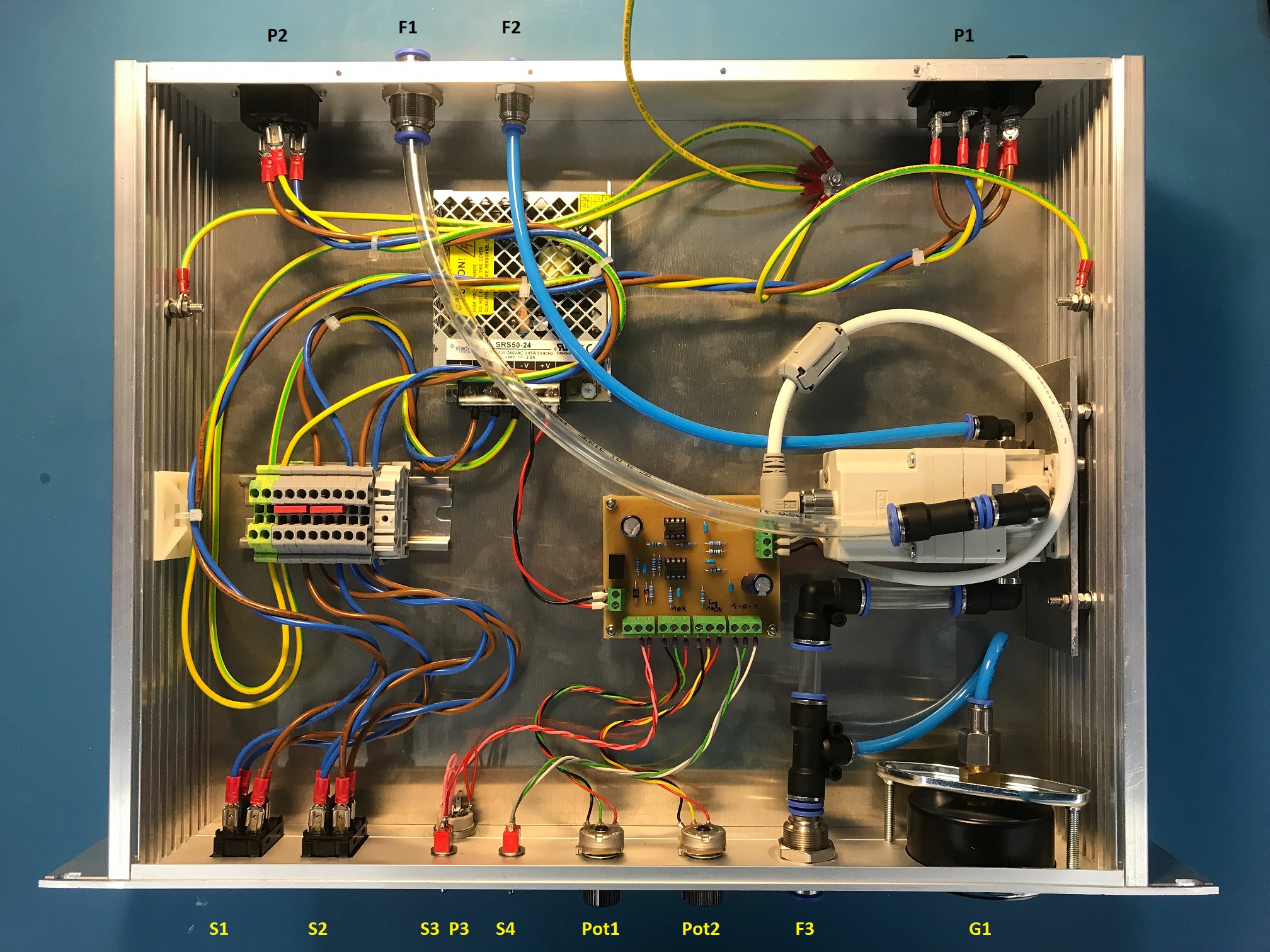

Supplement: Supplementary file 2 [file s-27-00360-sup2.zip › 09_SupMat9_VacuumControlUnit/01 SD wiring.JPG]

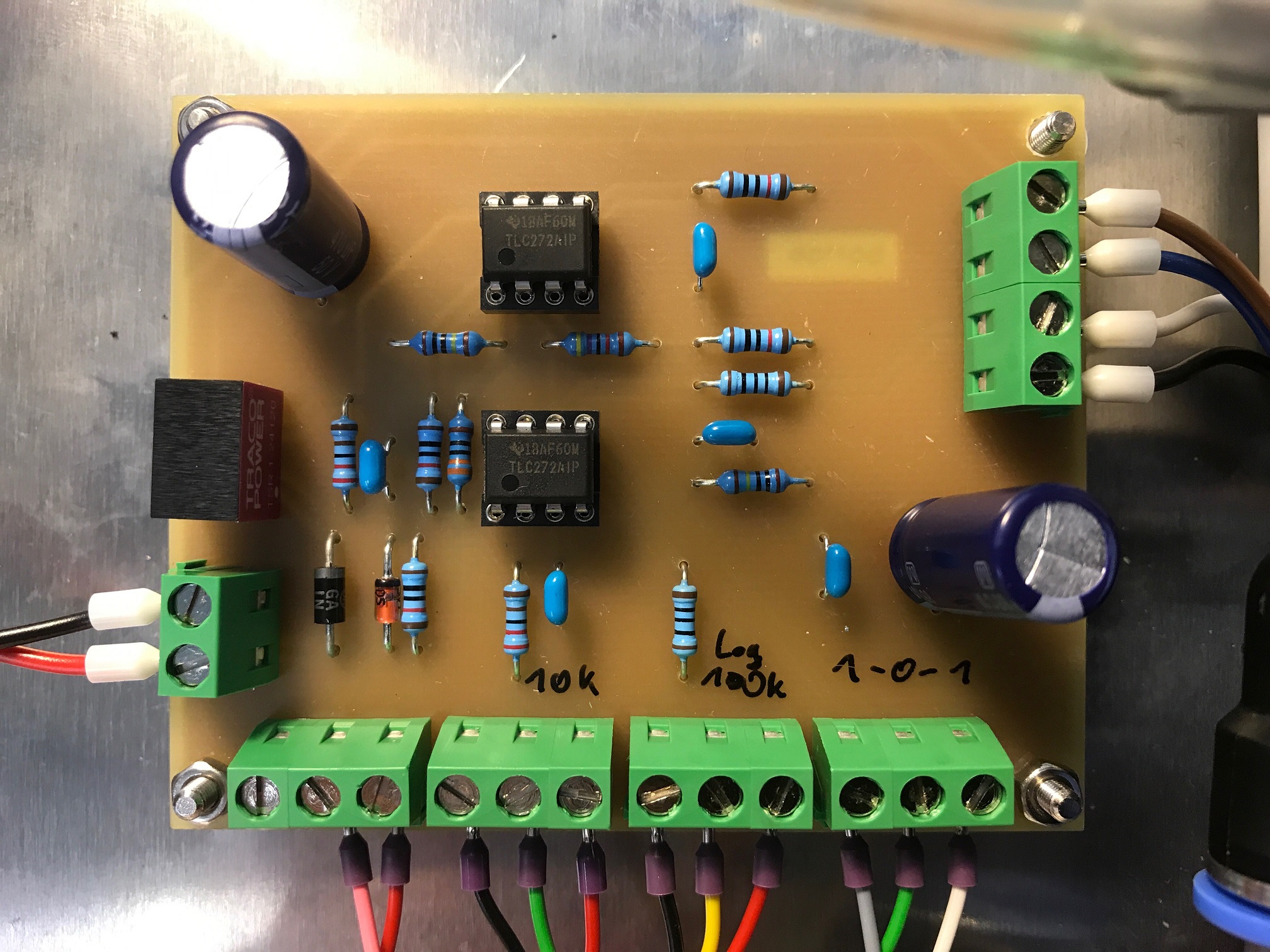

Supplement: Supplementary file 2 [file s-27-00360-sup2.zip › 09_SupMat9_VacuumControlUnit/02 PCB.JPG]

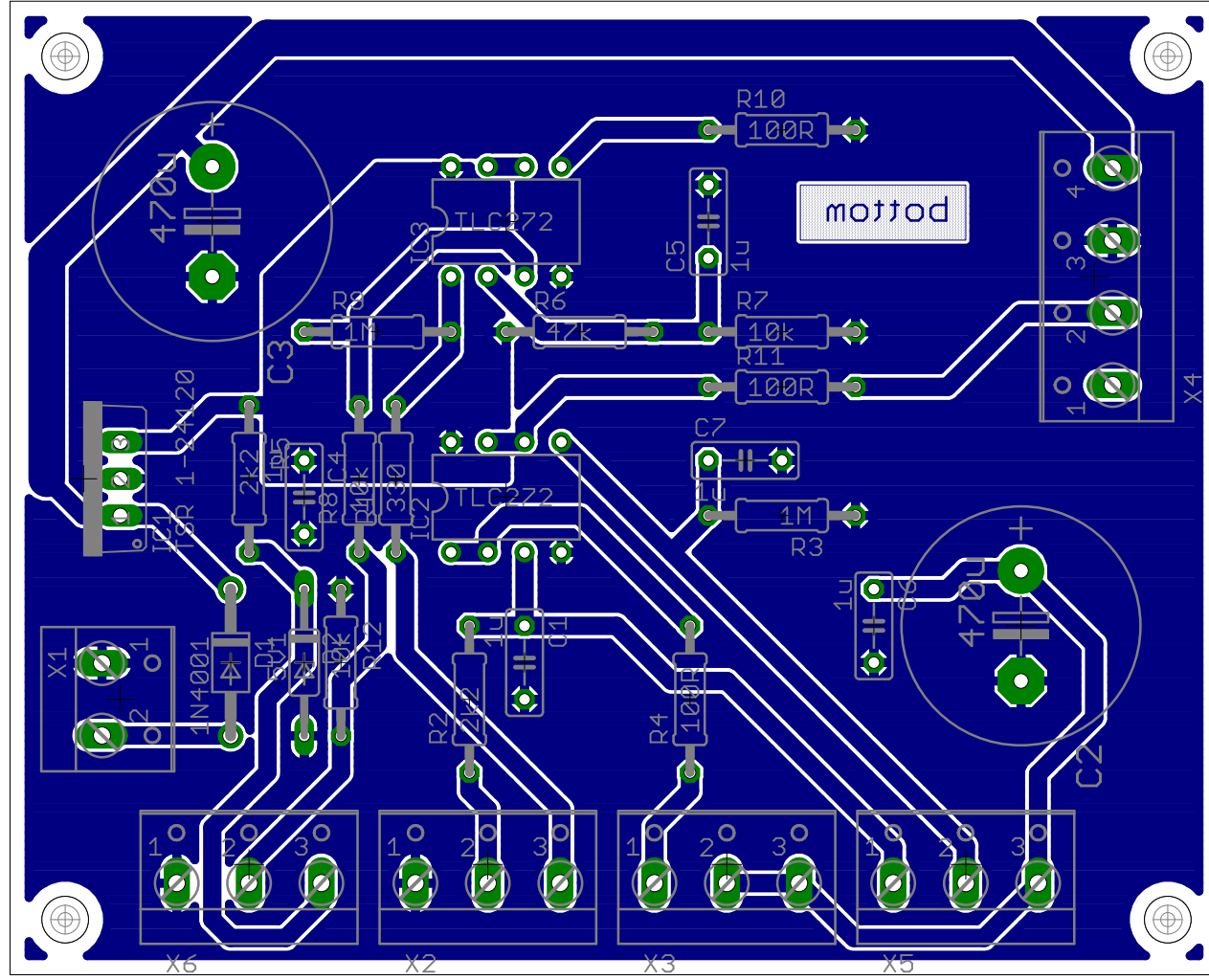

Supplement: Supplementary file 2 [file s-27-00360-sup2.zip › 09_SupMat9_VacuumControlUnit/electronics/PCB suction device board.pdf]

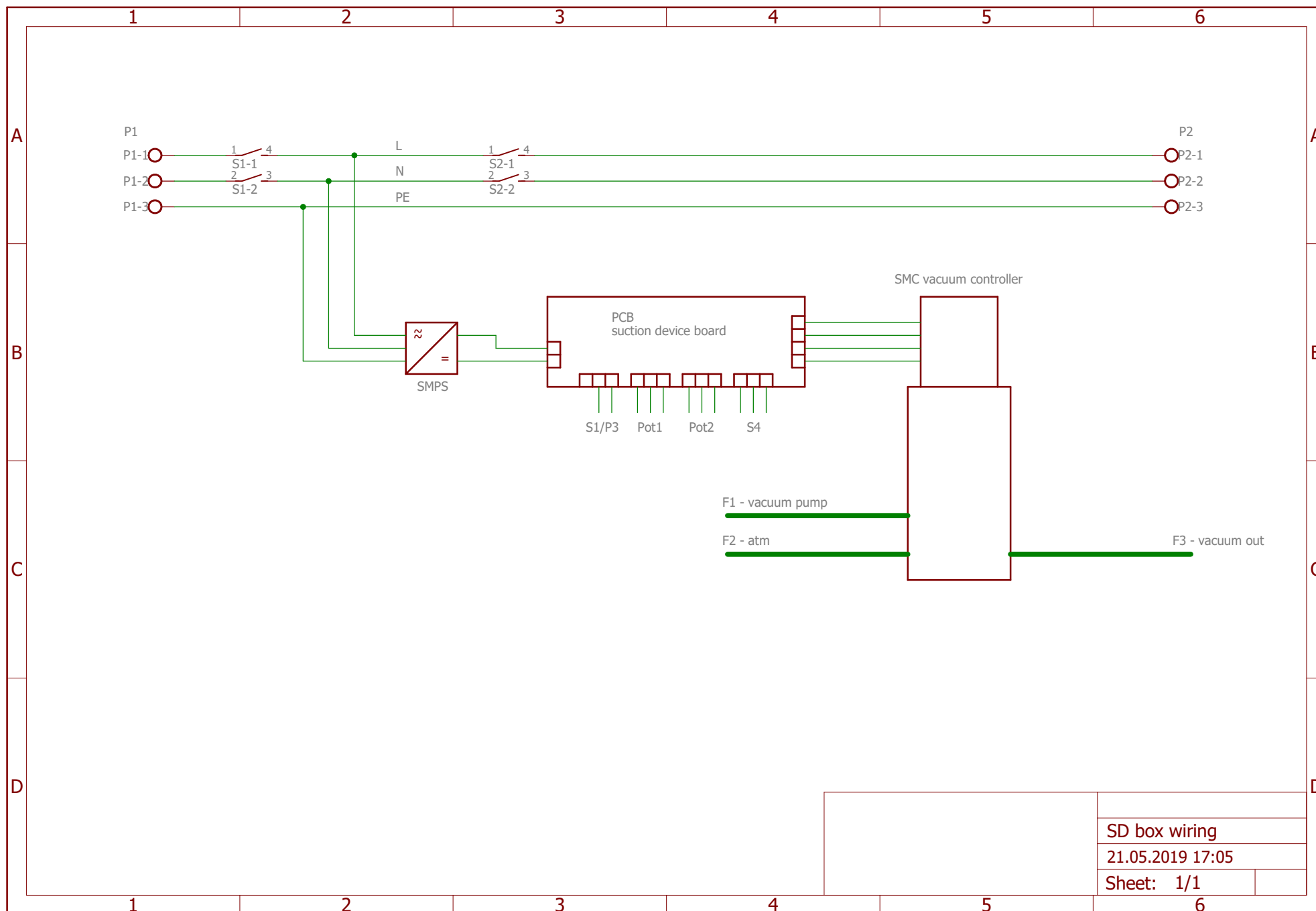

SD box wiring

21.05.2019 17:05

Sheet: 1/1

Supplement: Supplementary file 2 [file s-27-00360-sup2.zip › 09_SupMat9_VacuumControlUnit/electronics/SD box wiring.pdf]

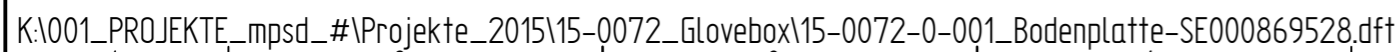

Supplement: Supplementary file 2 [file s-27-00360-sup2.zip › 10_SupMat10_humidityHood/15-0072-0-001_Bodenplatte-SE000869528.pdf]

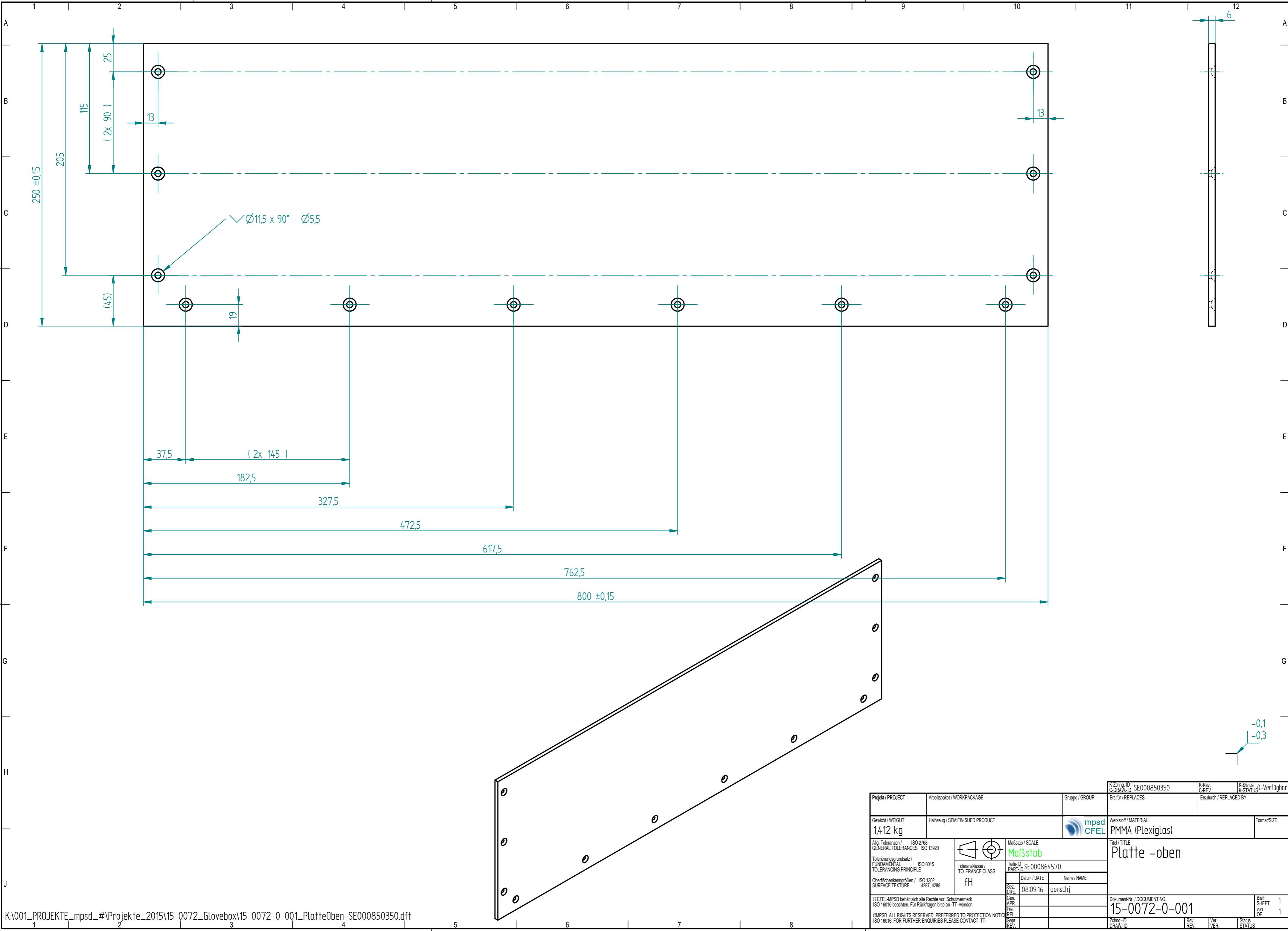

Supplement: Supplementary file 2 [file s-27-00360-sup2.zip › 10_SupMat10_humidityHood/15-0072-0-001_PlatteOben-SE000850350.pdf]

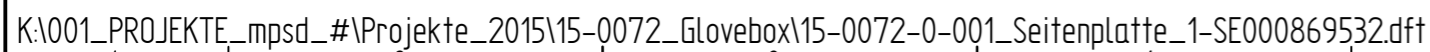

Supplement: Supplementary file 2 [file s-27-00360-sup2.zip › 10_SupMat10_humidityHood/15-0072-0-001_Seitenplatte_1-SE000869532.pdf]
