# Supplementary material for: The HARE chip for efficient time-resolved serial synchrotron crystallography
Source: J Synchrotron Radiat. 2020 Feb 27;27(Pt 2):360–70. doi: 10.1107/S1600577520000685 (PMC7064102; doi:10.1107/S1600577520000685)
Supplement: Supplementary file 2 [file Holder main.pdf › 02_SupMat2_holder/Holder main.pdf]

M:\00 SSU projects\SSU-MP0017 EMBL P14\4 Mechanics\20160919 Holder Magis\Holder main.dft

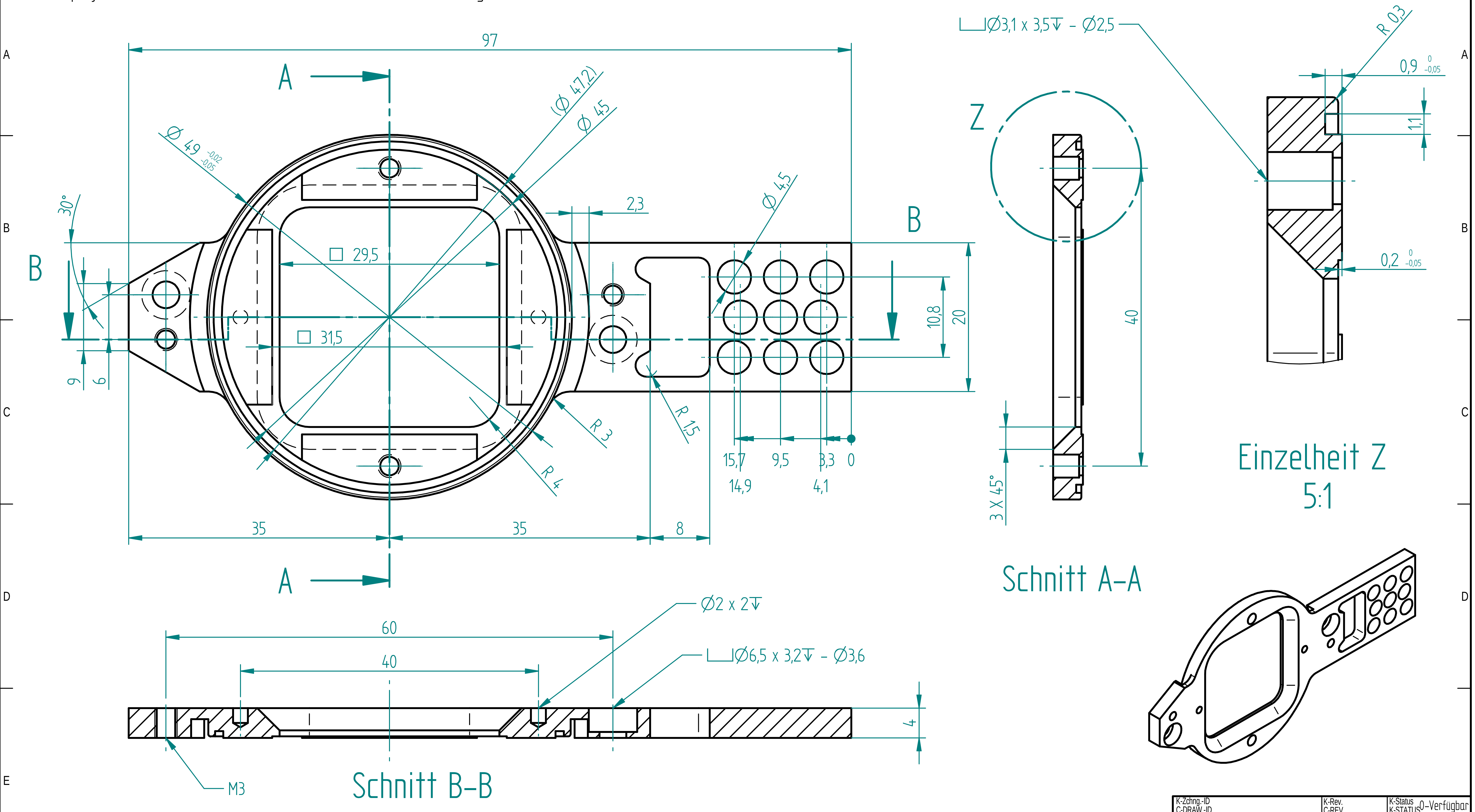


| Toleranz - klasse | Allgemeintoleranzen für Rechtwinkligkeit in mm bis 100 | über 100 bis 300 | über 300 bis 1000 | über 1000 bis 3000 |
|---|---|---|---|---|
| H | 0,2 | 0,3 | 0,4 | 0,5 |
| K | 0,4 | 0,6 | 0,8 | 1 |
| L | 0,6 | 1 | 1,5 | 2 |

| Toleranz - klasse | Allgemeintoleranzen für Geradheit und Ebenheit in mm bis 10 | über 10 bis 30 | über 30 bis 100 | über 100 bis 300 | über 300 bis 1000 | über 1000 bis 3000 |
|---|---|---|---|---|---|---|
| H | 0,02 | 0,05 | 0,1 | 0,2 | 0,3 | 0,4 |
| K | 0,05 | 0,1 | 0,2 | 0,4 | 0,6 | 0,8 |
| L | 0,1 | 0,2 | 0,4 | 0,8 | 1,2 | 1,6 |

| Toleranz - klasse | Grenzabmaße in mm für Nennmaßbereich in mm ( ISO 2768 ) bis 0,5 | über 0,5 bis 3 | über 3 bis 6 | über 6 bis 30 | über 30 bis 120 | über 120 bis 400 | über 400 bis 1000 | über 1000 bis 2000 | über 2000 bis 4000 | über 4000 bis 8000 |
|---|---|---|---|---|---|---|---|---|---|---|
| f (fein) | direkt am | ± 0,05 | ± 0,05 | ± 0,10 | ± 0,15 | ± 0,2 | ± 0,3 | ± 0,5 | - | - |
| m (mittel) | Nennmaß | ± 0,10 | ± 0,10 | ± 0,20 | ± 0,30 | ± 0,5 | ± 0,8 | ± 1,2 | ± 2 | ± 3 |
| g (grob ) | angeben | ± 0,15 | ± 0,20 | ± 0,50 | ± 0,80 | ± 1,2 | ± 2,0 | ± 3,0 | ± 4 | ± 5 |

K-Zchng.-ID / C-DRAW.-ID | K-Rev. / C-REV. | K-Status / K-STATUS: 0-Verfügbar

Projekt / PROJECT | Arbeitspaket / WORKPACKAGE | Gruppe / GROUP: Miller | Ers.für / REPLACES | Ers.durch / REPLACED BY

Gewicht / WEIGHT: 0,015 kg | Halbzeug / SEMIFINISHED PRODUCT | mpsd CFEL | Werkstoff / MATERIAL: EN AW–5083 (AlMg4,5Mn0,7) | Format/SIZE

Allg. Toleranzen / GENERAL TOLERANCES: ISO 2768, ISO 13920
Tolerierungsgrundsatz / FUNDAMENTAL TOLERANCING PRINCIPLE: ISO 8015
Oberflächenkenngrößen / SURFACE TEXTURE: ISO 1302, 4287, 4288
Toleranzklasse / TOLERANCE CLASS: mK

Maßstab / SCALE: 2 : 1
Teile-ID / PART-ID: SE001067330

Titel / TITLE: Holder Main

| | Datum / DATE | Name / NAME |
|---|---|---|
| Gez. / CRE. | 23.09.16 | tellkamf |
| Gen. / APR. | | |
| Frei. / REL. | | |
| Gepr. / REV. | | |

© CFEL-MPSD behält sich alle Rechte vor. Schutzvermerk ISO 16016 beachten. Für Rückfragen bitte an -TT- wenden

©MPSD. ALL RIGHTS RESERVED. PREFERRED TO PROTECTION NOTICE ISO 16016. FOR FURTHER ENQUIRIES PLEASE CONTACT -TT-

Dokument-Nr. / DOCUMENT NO.: 16–113–0–0001
Blatt / SHEET 1 von / OF 1
Zchng.-ID / DRAW.-ID | Rev. / REV. | Ver. / VER. | Status / STATUS
