# Supplementary material for: The HARE chip for efficient time-resolved serial synchrotron crystallography
Source: J Synchrotron Radiat. 2020 Feb 27;27(Pt 2):360–70. doi: 10.1107/S1600577520000685 (PMC7064102; doi:10.1107/S1600577520000685)
Supplement: Supplementary file 2 [file PCB suction device schematic.pdf › 09_SupMat9_VacuumControlUnit/electronics/PCB suction device schematic.pdf]

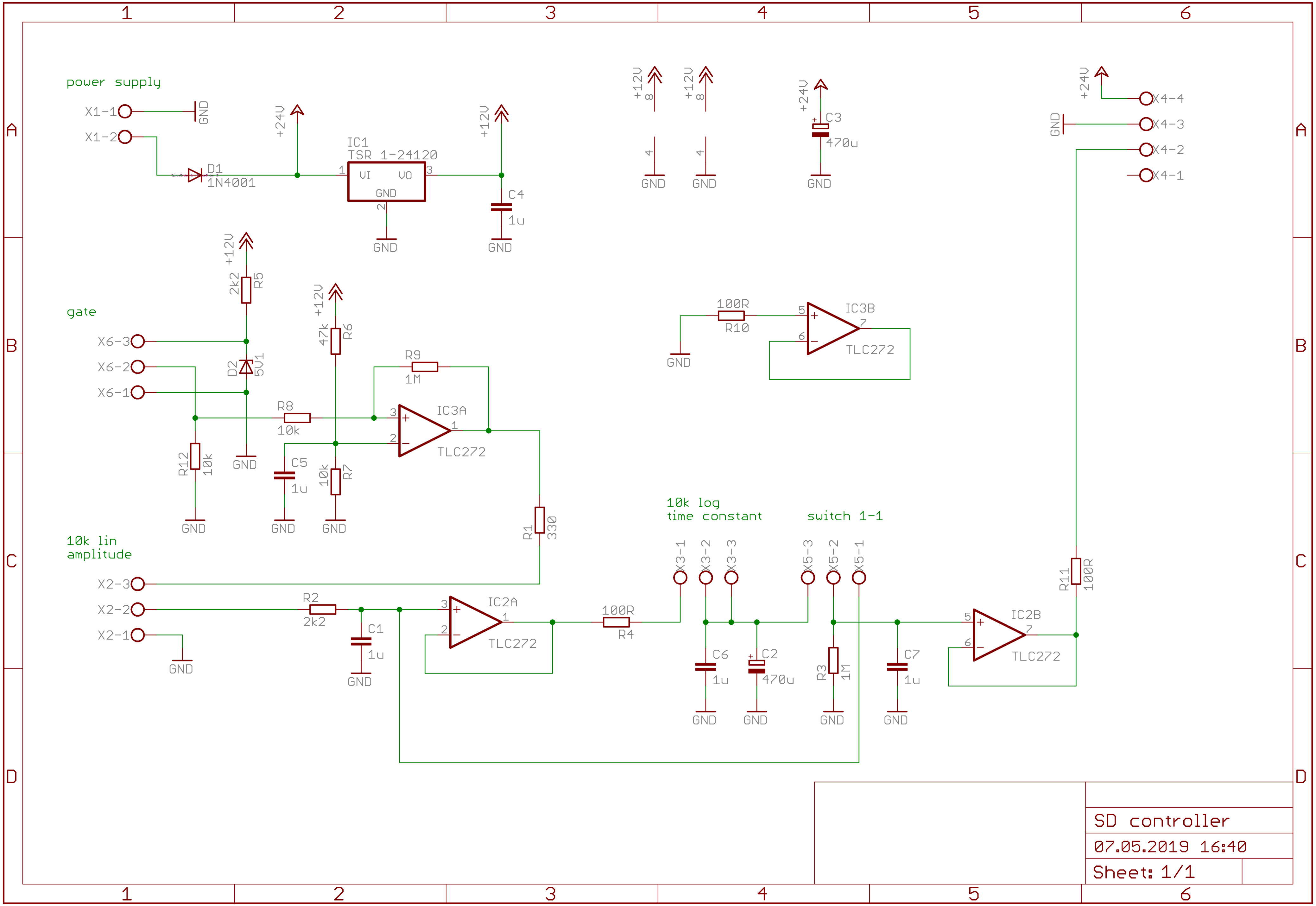
power supply
X1-1
X1-2
GND
D1
1N4001
+24V
IC1
TSR 1-24120
VI
VO
GND
+12V
C4
1u
gate
X6-3
X6-2
X6-1
R5
2k2
D2
5V1
R6
47k
R8
10k
R9
1M
IC3A
TLC272
R12
10k
C5
1u
R7
10k
R1
330
10k lin
amplitude
X2-3
X2-2
X2-1
R2
2k2
C1
1u
IC2A
TLC272
100R
R4
10k log
time constant
switch 1-1
X3-1
X3-2
X3-3
X5-3
X5-2
X5-1
C6
1u
C2
470u
R3
1M
C7
1u
IC2B
TLC272
R11
100R
R10
100R
IC3B
TLC272
C3
470u
X4-4
X4-3
X4-2
X4-1
SD controller
07.05.2019 16:40
Sheet: 1/1
